# Transcriptional network underpinning ploidy-related elevated leaf potassium in neo-tetraploids

**DOI:** 10.1093/plphys/kiac360

**Published:** 2022-08-05

**Authors:** Sina Fischer, Paulina Flis, Fang-Jie Zhao, David E Salt

**Affiliations:** Future Food Beacon of Excellence and the School of Biosciences, University of Nottingham, Nottingham, LE12 5RD, UK; Future Food Beacon of Excellence and the School of Biosciences, University of Nottingham, Nottingham, LE12 5RD, UK; State Key Laboratory of Crop Genetics and Germplasm Enhancement, College of Resources and Environmental Sciences, Nanjing Agricultural University, Nanjing, China; Future Food Beacon of Excellence and the School of Biosciences, University of Nottingham, Nottingham, LE12 5RD, UK

## Abstract

Whole-genome duplication generates a tetraploid from a diploid. Newly created tetraploids (neo-tetraploids) of Arabidopsis (*Arabidopsis thaliana*) have elevated leaf potassium (K), compared to their diploid progenitor. Micro-grafting has previously established that this elevated leaf K is driven by processes within the root. Here, mutational analysis revealed that the K^+^-uptake transporters K+ TRANSPORTER 1 (AKT1) and HIGH AFFINITY K+ TRANSPORTER 5 (HAK5) are not necessary for the difference in leaf K caused by whole-genome duplication. However, the endodermis and salt overly sensitive and abscisic acid-related signaling were necessary for the elevated leaf K in neo-tetraploids. Contrasting the root transcriptomes of neo-tetraploid and diploid wild-type and mutants that suppress the neo-tetraploid elevated leaf K phenotype allowed us to identify a core set of 92 differentially expressed genes associated with the difference in leaf K between neo-tetraploids and their diploid progenitor. This core set of genes connected whole-genome duplication with the difference in leaf K between neo-tetraploids and their diploid progenitors. The set of genes is enriched in functions such as cell wall and Casparian strip development and ion transport in the endodermis, root hairs, and procambium. This gene set provides tools to test the intriguing idea of recreating the physiological effects of whole-genome duplication within a diploid genome.

## Teaser

Whole-genome duplication leads to immediate effects on leaf potassium levels, which is connected to changes in a transcriptional network of 92 genes.

## Introduction

It is well known that whole-genome duplication events occurred multiple times throughout land plant evolution, suggesting an evolutionary benefit (Alix et al., 2017). However, newly generated polyploids face severe problems, such as maintaining faithful chromosome separation during meiosis, or changes in cell architecture resulting from increased chromatin content (Comai, 2005). The short-term survival of neo-tetraploids was, therefore, speculated to occur at times of environmental change which would adversely affect diploid progenitors, neutralizing their evolutionary advantage of being locally adapted (Van de Peer et al., 2017). Neo-tetraploid Arabidopsis (*Arabidopsis thaliana*), rice (*Oryza sativa*), and citrange (*Citrus sinensis* L. Osb. ×*Poncirus trifoliata* L. Raf.) all have increased tolerance to salinity and drought as a result of whole-genome duplication, suggesting possible short-term survival benefits of whole-genome duplication (Chao et al., 2013; Pozo and Ramirez‐Parra, 2014; Yang et al., 2014; Ruiz et al., 2016; Wang et al., 2021). Leaf potassium (K) concentrations are higher in neo-tetraploid Arabidopsis compared to their diploid progenitor, driven by processes in the root as determined by micro-grafting (Chao et al., 2013). This suggests one possible mechanism whereby whole-genome duplication can enhance salinity tolerance; through the enhanced ability of neo-tetraploids to maintain K^+^/Na^+^ homeostasis under salinity stress (Munns and Tester, 2008). Consistent with this, tetraploid rootstock-grafted watermelon (*Citrullus lanatus*) plants are more tolerant to salt stress than are diploid plants, and tetraploids also maintain better K^+^/Na^+^ homeostasis (Zhu et al., 2018). Similar results have also been observed for tetraploid rice with elevated salinity tolerance and reduced accumulation of Na (Wang et al., 2021). Neo-tetraploid Arabidopsis and rice also show enhanced abscisic acid (ABA) and jasmonic acid (JA) signaling, respectively, again consistent with constitutive abiotic stress tolerance driven by whole-genome duplication (Pozo and Ramirez‐Parra, 2014; Wang et al., 2021). These observations suggest that under adverse environmental conditions, the negative impacts of whole-genome duplication can be overcome, providing a positive fitness benefit. Although improved tolerance to abiotic stresses in polyploids has been documented, the exact molecular processes underlying these responses still remain to be discovered (Fox et al., 2020).

To start to address this knowledge gap, and identify the molecular mechanisms linking whole-genome duplication to improved abiotic stress tolerance, we focused on the ability of neo-tetraploids to better maintain K^+^/Na^+^ homeostasis under salinity stress. We investigated the mechanism driving the previously observed difference in leaf K between neo-tetraploid Arabidopsis and their diploid progenitor (Chao et al., 2013) caused by whole-genome duplication. Our main approach was to select genetic mutants with defects in target processes, and contrast the leaf K between the diploid mutant and the neo-tetraploid mutant generated after whole-genome duplication of the diploid mutant. This approach compares genetically identical plants with diploid or tetraploid genomes lacking any functional copies of the gene(s) of interest. It has been established that processes within the root (Chao et al., 2013) drive elevated leaf K of neo-tetraploid Arabidopsis compared to their diploid progenitor. We therefore tested the hypothesis that elevated activity of root K^+^-uptake transporters in neo-tetraploids is responsible for the difference in leaf K between neo-tetraploids and their progenitor diploids. Additionally, we also selected a broader set of 38 candidate genes affecting various processes previously implicated in K homeostasis. We obtained mutants and transgenic lines of these genes, and tested the impact of these genes on the elevated leaf K in neo-tetraploids caused by whole-genome duplication of the progenitor diploids. From this series of genetic tests, we identified three genes that when disrupted suppress the elevated leaf K in neo-tetraploids caused by whole-genome duplication of the progenitor diploids. Finally, we compared the root transcriptome of wild-type neo-tetraploid and diploid progenitor to identify all differentially expressed genes (DEGs) due to whole-genome duplication in roots. Importantly, we further refined this selection to just those DEGs in roots associated with the difference in leaf K between neo-tetraploid and diploid progenitor. To do this, we removed those genes that we found to be still differentially expressed between neo-tetraploid and diploid in roots of the mutants we had identified that suppress the difference in leaf K between neo-tetraploid and diploid progenitor. Using this approach, we defined a core set of 92 genes that provide functional insight into the underlying molecular mechanisms linking whole-genome duplication and elevated leaf K in neo-tetraploids compared to their diploid progenitors. This gene set provides the tools needed to test the hypothesis that it is possible to recreate the physiological effects of whole-genome duplication within a diploid genome, leading the way to potential mechanisms for improving abiotic stress tolerance via recreating tetraploid-like phenotypes within a diploid genome.

## Results and discussion

### Impact of K^+^-uptake transporters on elevated leaf K in neo-tetraploids

**Figure 1:**
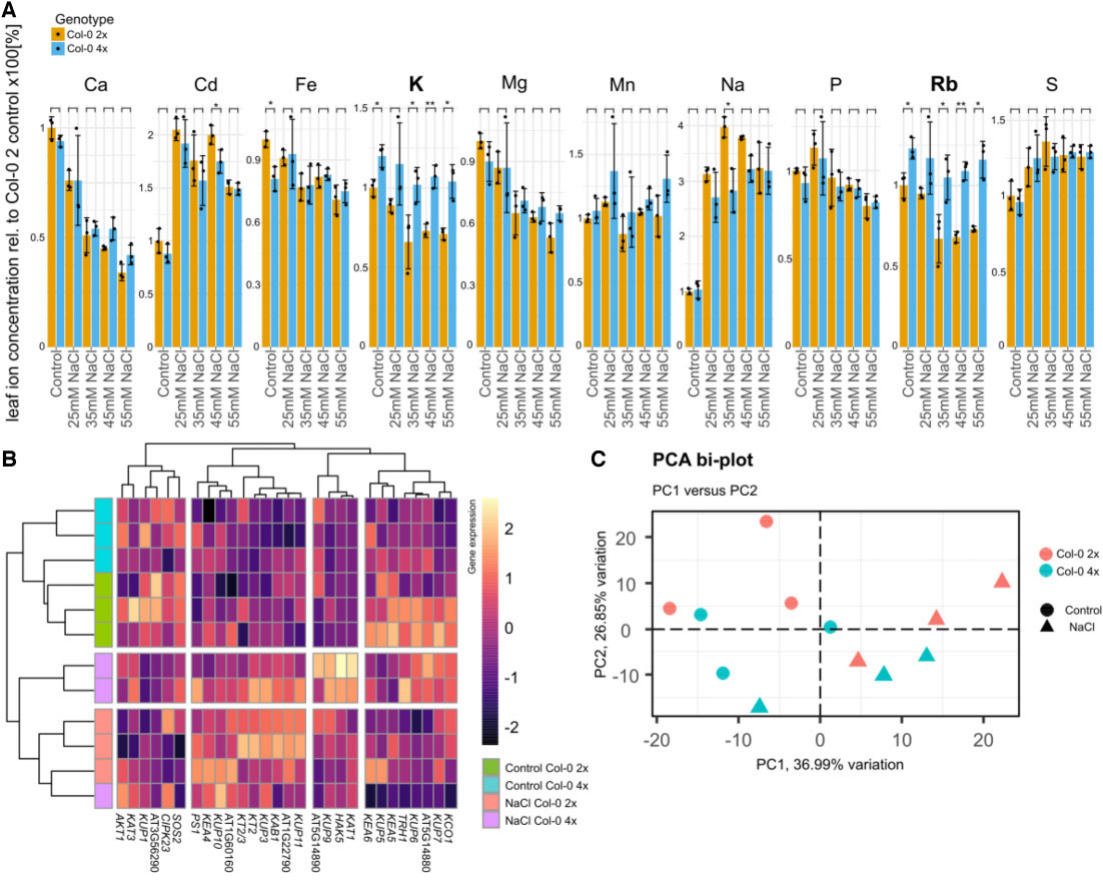
Ploidy-dependent ionomic differences and K transporters in neo-tetraploid Arabidopsis. A, The leaf ionome of diploid and neo-tetraploid Arabidopsis wild-type (Col-0) plants was assessed in response to varying salt stress. The bar plots show ion concentrations relative to those of Col-0 under control conditions. Plants were grown on agar solidified 1/4 Hoagland medium for 7 days. *n* = 3, A two-way ANOVA for genotype and treatment revealed significant differences in K and Rb due to ploidy (diploid 2x, neo-tetraploid 4x) (Supplemental Table S1). Following this a *t* test shows significance differences between pairs: ^*^P ≤ 0.05, ^**^P ≤ 0.01, ^***^P ≤ 0.001, and ^****^P ≤ 0.0001, error bars as sd. B, RNAseq derived gene expression pattern of 28 of 31 K genes taken from Thalemine (using the keyword search “K transporter” and restricting it to the 31 with the highest scores) was measured in roots of diploid and neo-tetraploid wild-type. Color scale shows centered gene expression across all samples and genes. Plants were grown on 1/4 Hoagland’s medium containing sucrose and agar with or without 35 mM NaCl. A cluster analysis of samples from roots of diploid and tetraploid wild-type plants shows a grouping based on ploidy. Genes with higher expression are shown with brighter colors. The annotation column identifies treatment and genotype. C, A PCA analysis of the same set of genes shows PC1 correlated with treatment and PC2 correlated with ploidy. The legend indicate differences in ploidy and different symbols distinguish different treatments. Together they explained the majority (>63.84%) of the variation in expression of the 31 K-genes.

As previously reported, the difference in leaf K concentration in neo-tetraploids compared to their diploid progenitor, where neo-tetraploids accumulate increased leaf K, is dependent on the ploidy status of the root (Chao et al., 2013). We therefore tested the hypothesis that increased activity of K^+^-uptake transporters in the roots of neo-tetraploids is responsible for the elevated leaf K in neo-tetraploids compared to their diploid progenitor. We grew neo-tetraploid and diploid wild-type plants on agar-solidified media with and without added NaCl. We quantified the leaf ionome of the plants (Figure 1A). In line with the previous results (Chao et al., 2013), a two-way analysis of variance (ANOVA) analysis of the data revealed significantly (*P* = 0.045; Supplemental Table S1) elevated leaf K concentration in neo-tetraploids compared to their diploid progenitor, and also the K chemical analog rubidium (Rb), present in trace amounts in the growth medium (*P* = 0.018; Supplemental Table S1) (Figure 1A, absolute concentrations of ions per dry weight of plant tissue can be found in Supplemental Table S2). These increases were observed for all levels of NaCl treatment. Furthermore, neo-tetraploids have significantly reduced accumulation of Na in leaves at 35- and 45-mM NaCl concentration (*P* < 0.001 and *P* = 0.041, respectively, Supplemental Table S1; Figure 1A), as previously observed in neo-tetraploid rice (Wang et al., 2021). No other mineral nutrients show a significant increase in leaves between neo-tetraploid and diploid progenitor under control conditions, or a consistent pattern of increase in neo-tetraploids after NaCl treatment (Supplemental Table S1; Figure 1A). To determine if genes involved in K^+^ uptake are differentially expressed in roots of neo-tetraploids compared to diploid progenitor, we performed an RNA-Seq experiment on roots of both wild-type neo-tetraploid and diploid grown in the absence and presence of 35 mM added NaCl. We hypothesized that the K phenotype could be due to K transporter genes which are differentially expressed between diploids and neo-tetraploid roots. We selected a set of genes representative of known K transporters (genes identified at Thalemine https://bar.utoronto.ca/thalemine/begin.do, top hits) and performed a hierarchical clustering of our transcriptome data to show the expression pattern of this set of known K transporter genes in roots of both diploids and neo-tetraploids (Figure 1B). The impact of salinity stress had overall the largest effect on the expression of the K transport genes, but within each treatment, gene expression was clearly differentiated between diploids and neo-tetraploids. Likewise, a principal component analysis (PCA) using the K transporter genes showed that 64% of the variation in gene expression can be explained by the two factors of salinity stress (37%) and ploidy (27%) (Figure 1C).

**Figure 2:**
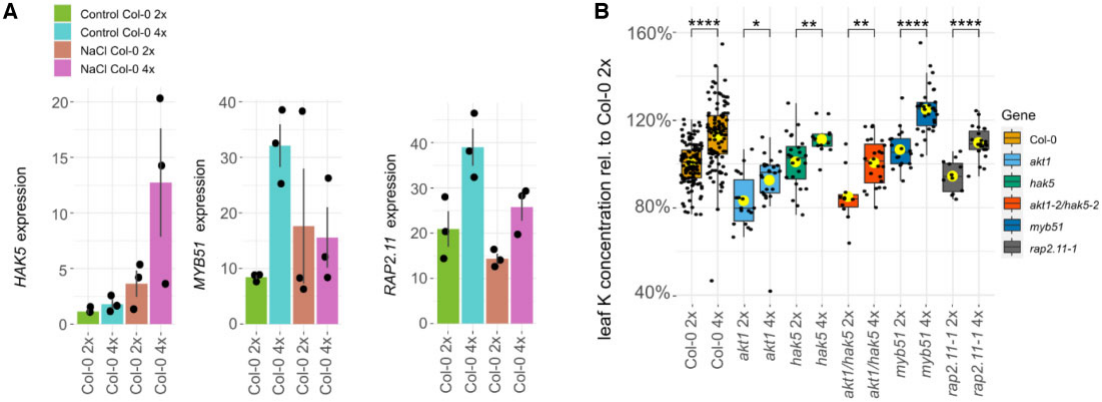
Role of the main K uptake transporters on increased K uptake in neo-tetraploid Arabidopsis. A, Bar plots show *HAK5, MYB51, and RAP2.11* expression as analyzed by RNA-Seq (*n* = 3, individual samples, error bars in se) in roots of diploid and neo-tetraploid wild-type plants. DEGs were defined as having a fold change of >2 and a diverge probability of >0.8 in RNAseq analysis. B, Boxplot shows leaf K concentration in diploids and their neo-tetraploid counterparts normalized to diploid wild-type. The center line shows the median, box limits represent the first and third quartiles (the 25th and 75th percentiles), upper and lower whiskers extend to larges or smallest value no further than 1.5 interquartile range and dots beyond whiskers show outliers. Individual values are plotted as dots. *n* = 11–121 individual samples, data from two independent experiments using peat-based soil in either jiffies or larger pots. ANOVA (Supplemental Table S1) showed significant differences between diploids and neo-tetraploids. A *t* test shows differences between diploids and neo-tetraploid pairs. ^*^P ≤ 0.05, ^**^P ≤ 0.01, ^***^P ≤ 0.001, and ^****^P ≤ 0.0001 Yellow dot: averages. Diploid (2x), neo-tetraploid (4x).

To identify a K transporter which could contribute to the K phenotype, we determined those genes that are statistically differentially expressed in roots between neo-tetraploid and diploid in our RNAseq data. These were termed as DEGs (Supplemental Figure S1; Supplemental Table S3). Among the K transporters (Figure 1B), only the gene *High Affinity K Transporter 5* (*HAK5*) was differentially expressed. We also compared these DEGs with a set of genes known to be regulated in response to low external concentrations of K (Ahn et al., 2004; Pyo et al., 2010; Rubio et al., 2010; Ragel et al., 2015; Nieves‐Cordones et al., 2019). Of these known low K response genes *HAK5*, two known *HAK5* regulators *MYB domain protein 51 (MYB51*) and *Related to AP2 11* (*RAP2.11*) (Hong et al., 2013; Nieves-Cordones et al., 2014) were found to be differentially expressed in roots between neo-tetraploids and their diploid progenitor (Figure 2A; Supplemental Figures S2 and S3). *HAK5* shows a two-fold increase in expression in neo-tetraploids compared to their diploid progenitor under control conditions, and this increases to a four-fold difference after treatment with 35-mM NaCl (Figure 2A). *MYB51* expression is only elevated in neo-tetraploids in the absence of NaCl, while *RAP2.11* expression shows a two-fold increase between neo-tetraploids and diploids regardless of 35-mM NaCl treatment. We concluded that, based on its expression, neo-tetraploid Arabidopsis plants may be sensing an internal K deficiency and are inducing expression of *HAK5* via *RAP2.11* and *MYB51*. We thus decided to investigate the effect of *HAK5* in neo-tetraploids. Arabidopsis has two major K^+^-uptake systems, *HAK5* and *K^+^ Transporter 1* (*AKT1*)*.* HAK5 is the dominant uptake system at lower concentrations of K^+^ up to 30 µM, and is transcriptionally and posttranscriptionally regulated by K^+^ (Gierth et al., 2005; Rubio et al., 2008; Nieves-Cordones et al., 2010; Ragel et al., 2015). AKT1 is able to contribute and even compensate for the lack of HAK5 completely at 10 µM external K^+^, while at concentrations of >500 µM, AKT1 becomes the main pathway for K^+^-uptake (Hirsch et al., 1998; Spalding et al., 1999; Qi et al., 2008; Rubio et al., 2008, 2010; Pyo et al., 2010; Nieves‐Cordones et al., 2019). AKT1 is primarily regulated posttranscriptionally (Wang et al., 2010; Zhang et al., 2015). The elevated expression of *HAK5* and its regulators *MYB51* and *RAP2.11* in neo-tetraploids supports the hypothesis that elevated leaf K in neo-tetraploids compared to diploid progenitor is driven by elevated activity of root K^+^-uptake system (s). To test this hypothesis, we assessed the impact of a lack of both major K^+^-uptake systems AKT1 and HAK5 on the difference in leaf K concentration in neo-tetraploids compared to their diploid progenitor. We obtained knockout mutants for *AKT1*, and *HAK5* and its regulators *MYB51* and *RAP2.11*, in diploid backgrounds, and we doubled the genomes of each to produce neo-tetraploids lacking all functional copies of each of the genes. To account for redundancy between the AKT1 and HAK5 transporters, we also generated the neo-tetraploid of the *akt1-2 hak5-2* double mutant (Ragel et al., 2015) which lacks all functional copies of both genes. After growing the plants in soil, we assessed the leaf ionome in each genotype for both diploids and neo-tetraploids (Figure 2B; Supplemental Figure S4). A two-way ANOVA of the leaf K concentrations (Supplemental Table S1) established that ploidy has a significant impact on leaf K (i.e. diploid and neo-tetraploid, *P* < 0.001), as we observed when plants were grown on agar-solidified nutrient media (Figure 1A; *P* = 0.045; Supplemental Table S1). The two-way ANOVA also established that *akt1* and *akt1hak5* double mutant show significantly reduced (Figure 2A; *P* < 0.001; Supplemental Table S1) leaf K in both the diploid and neo-tetraploid compared to wild-type, as expected, as AKT1 is the dominant K^+^-uptake system under our growth conditions. The *hak5* mutant showed no significant difference (Figure 2B, *P* = 0.667; Supplemental Table S1) in leaf K compared to wild-type, for both neo-tetraploid and diploid. This is expected as HAK5 does not play a dominant role in K^+^ uptake under our growth conditions. Further, loss of function of the *HAK5* regulators *MYB51* and *RAP2.11* does not reduce leaf K either in neo-tetraploid or diploid progenitor, with *myb51* actually showing a slight but significant increase in leaf K (Figure 2B; *P* = 0.024; Supplemental Table S1). The two-way ANOVA also established that there is no overall significant interaction between ploidy and gene (*P* = 0.5542; Supplemental Table S1). This test for ploidy * gene interaction assesses if the established significant elevation of leaf K due to whole-genome duplication in neo-tetraploids is affected by loss of function of any of the tested genes. The two-way ANOVA established that the loss of function of *AKT1*, or *HAK5* and its regulators *MYB51* and *RAP2.11*, does not affect the significant difference in leaf K between neo-tetraploids and their progenitor diploids (Figure 2B). Leaf K is elevated in neo-tetraploids compared to diploid progenitor regardless of the presence or absence of *AKT1* or *HAK5*. We conclude that increased activity of AKT1 or HAK5 in neo-tetraploids cannot explain the differential increase in leaf K after whole-genome duplication in neo-tetraploids compared to the diploid progenitor. We make similar observations for leaf Rb, a chemical analog of K (Supplemental Figure S4 and Supplemental Table S1). We also observed smaller variations in the concentration of some other mineral nutrients between neo-tetraploids and diploids but with no clear patterns (Supplemental Figure S4).

### Exploring a broader set of processes impacting elevated leaf K in neo-tetraploids

**Figure 3:**
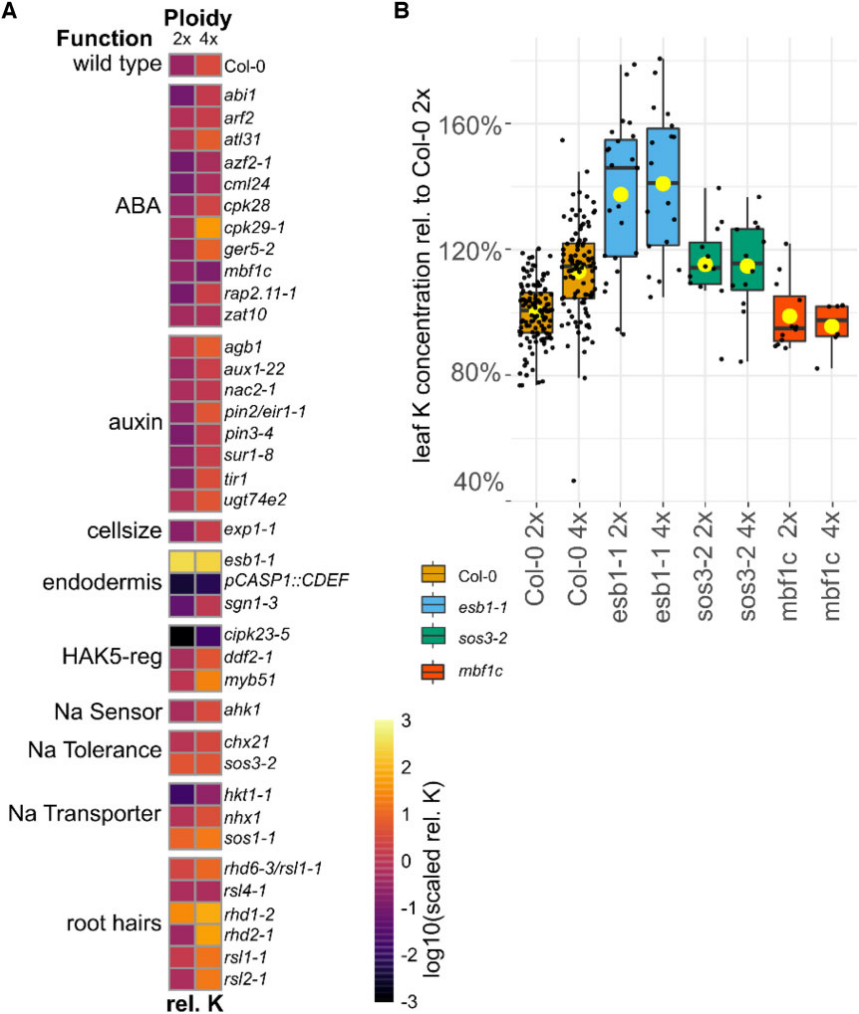
Selected candidates genes. A, Heatmap displays K concentration in leaves of diploid and neo-tetraploid mutants grown on soil relative to the K concentration in diploid wild-type plants. The color scale shows genotypes with lower (darker) and higher (brighter) than average relative K concentrations within each functional group annotated using previously published studies. rel.: relative; HAK5-reg: High Affinity K Transporter 5 regulators. B, Boxplot shows the leaf K concentration relative to diploid wild-type of the three mutants, esb1-1, mbf1c, and sos3-2 for which a significant interaction between ploidy and gene was found in the two-way ANOVA (Supplemental Table S1). The center line shows the median, box limits represent the first and third quartiles (the 25th and 75th percentiles), upper and lower whiskers extend to larges or smallest value no further than 1.5 interquartile range and dots beyond whiskers show outliers. Individual values are plotted as dots. *n* = 6–1,279, Yellow dot: averages. Diploid (2x), neo-tetraploid (4x).

With differential activity of *AKT1* and *HAK5* between neo-tetraploids and diploids ruled out as a possible driver of the difference in leaf K between neo-tetraploids and diploid progenitors, and no further differentially expressed K transporters genes, we explored other possible genes involved in the difference between leaf K in neo-tetraploids and their diploid progenitors. We selected 38 genes based on the published literature that have the potential to impact K homeostasis. These selected candidate genes were grouped, based on their function, into nine clusters (Figure 3). We obtained loss-of-function mutants in diploid backgrounds for each of these genes (except *pCASP1::CDEF1* which is a transgenic line), and through whole-genome duplication generated neo-tetraploids which also lack each of the genes (or express *pCASP1::CDEF1*). These lines were grown in soil and the leaf ionome assessed. Figure 3A shows the leaf K concentration relative to that of the diploid wild-type for all these lines, grouped by function. A two-way ANOVA revealed that overall ploidy had a significant impact on leaf K (*P* = <2e-16; Supplemental Table S1). The two-way ANOVA also revealed that gene had a significant impact on leaf K (*P* < 2e-16; Supplemental Table S1), as might be expected given these genes were selected as potentially impacting K homeostasis. We observe significant reductions in leaf K in lines *cipk23* (Ragel et al., 2015) and *pCASP1::CDEF1* (Barberon et al., 2016), and significant increases in leaf K in *rhd1*, *rhd6/rsl1* (Ahn et al., 2004), salt overly sensitive (*sos)1*, *sos3*, and *esb1* (Baxter et al., 2009) (Supplemental Table S1), as has been previously observed, in some cases. The two-way ANOVA also identified significant ploidy × gene interaction for the pairwise comparisons ploidy[4x] * gene[*ESB1*] (*P* = 0.003), ploidy[4x] * gene[*SOS3*] (*P* = 0.02), ploidy [4x] * gene [*MBF1c*] (*P* = 0.019), and ploidy[4x] * gene[*RHD2*] (*P* = 0.038). For *esb1-1, sos3-2*, and *mbf1c*, the increased leaf K concentration normally observed in wild-type neo-tetraploids compared to the diploid progenitor is suppressed, while in *rhd2* this difference is enhanced. For the three mutants that suppress the elevated leaf K in neo-tetraploids, *esb1-1*, *sos3-2*, and *mbf1c* (Figure 3b), a two-way ANOVA shows both factors, ploidy and gene, as well as their interaction now to be significant in affecting the leaf K concentration (*P* = 1.28e-10, <2.2e-16, and 0.003088, respectively, Supplemental Table S1). Both *esb1-1* and *sos3-1* significantly increase leaf K (*P* < 0.001) in both diploid and neo-tetraploid compared to wild-type, while *mbf1c* did not have an impact on leaf K concentrations compared to wild-type (*P* = 0.613). All three mutations also suppress the increased leaf K normally observed between neo-tetraploids and diploid progenitor (*P* = 0.026, 0.019, 0.012, respectively, Supplemental Table S1; Figure 3B).

*SOS3* (also known as *CBL4*) encodes a Ca sensor which operates in the SOS signaling pathway in response to salinity stress (Gong et al., 2004). SOS3 regulates Na^+^ efflux, leading to reduced K^+^ efflux through depolarization-activated K^+^ Outward Rectifying channels (Assaha et al., 2017). Elevated leaf K in neo-tetraploids compared to diploid progenitors may require SOS signaling to enable enhanced K accumulation, even at low external Na concentrations.

*ESB1* on the other hand is an important component for the biosynthesis of Casparian strips, which form in the cell wall at the endodermis (Baxter et al., 2009). In *esb1-1* Casparian strips are disturbed, leading to an increase in leaf K concentrations. It is thought that this increase in leaf K is due to the enhanced endodermal suberization observed in this mutant (Baxter et al., 2009). Increasing the suberized zone of the endodermis prevents K leaking from the stele where it is more highly concentrated than in the surrounding root tissue (Barberon et al., 2016). Neo-tetraploid *esb1-1* does not have elevated leaf K when compared to the diploid progenitor, unlike wild-type where we observe elevated leaf K in the neo-tetraploid compared to its diploid progenitor. This suggests that a normally functioning endodermis plays an important role in the enhanced leaf K concentration we observe in wild-type neo-tetraploids.

*MBF1c* is one of three *MBF* genes in Arabidopsis. MBFs are transcriptional co-activators (Takemaru et al., 1998). They are highly conserved among eukaryotes (Alavilli et al., 2017). In Arabidopsis, *MBF1a* and *1b* show high sequence similarities and expression profiles (Tsuda et al., 2004). They are thought to be important for pathogen response. *MBF1c* is distinct from *a* and *b*. It is induced by ABA treatment and has been shown to be involved in mediating salinity, osmotic, cold, and heat stress (Suzuki et al., 2005; Alavilli et al., 2017). In neo-tetraploids *MBF1c* shows higher expression in roots only in plants grown under control conditions (Supplemental Figure S1). Upon Na treatment *MBF1c* is induced in roots of wild-type diploid plants. However, in neo-tetraploids *MBF1c* is constitutively activated to the levels seen in the Na-treated diploids, with Na treatment having no further impact on expression in neo-tetraploids. Identification of genes induced in an *MBF1c* ectopic over expression line revealed potential targets for co-regulation through MBF1c (Suzuki et al., 2005). Among the 87 DEGs in the *MBF1c* over expression line, 18 are identified here as DEGs in roots of wild-type neo-tetraploids when grown under control conditions from our RNA-Seq experiment, supporting a role for MBF1c in the transcriptional response of roots to whole-genome duplication. Multiprotein binding factor1 (MBF1c) sits upstream of several ABA and ethylene response genes (Tsuda et al., 2004; Suzuki et al., 2005), and we have established that it is necessary for the elevated leaf K observed in wild-type neo-tetraploids (Figure 2B). However, other genes known to be directly involved in ABA signaling such as *ABI1*, whose mutant *abi1-1* is insensitive to ABA (Koornneef et al., 1982), are not required for elevated leaf K in neo-tetraploids, as loss-of-function mutants in these genes still showed elevated leaf K in neo-tetraploids (ploidy[4x] * gene[*ABI1*] *P* = 0.645, Supplemental Table S1; Figure 3A). This suggests that there is not a direct link between ABA signaling and elevated leaf K in neo-tetraploids.

### Root transcriptional network associated with elevated leaf K in neo-tetraploids

**Figure 4:**
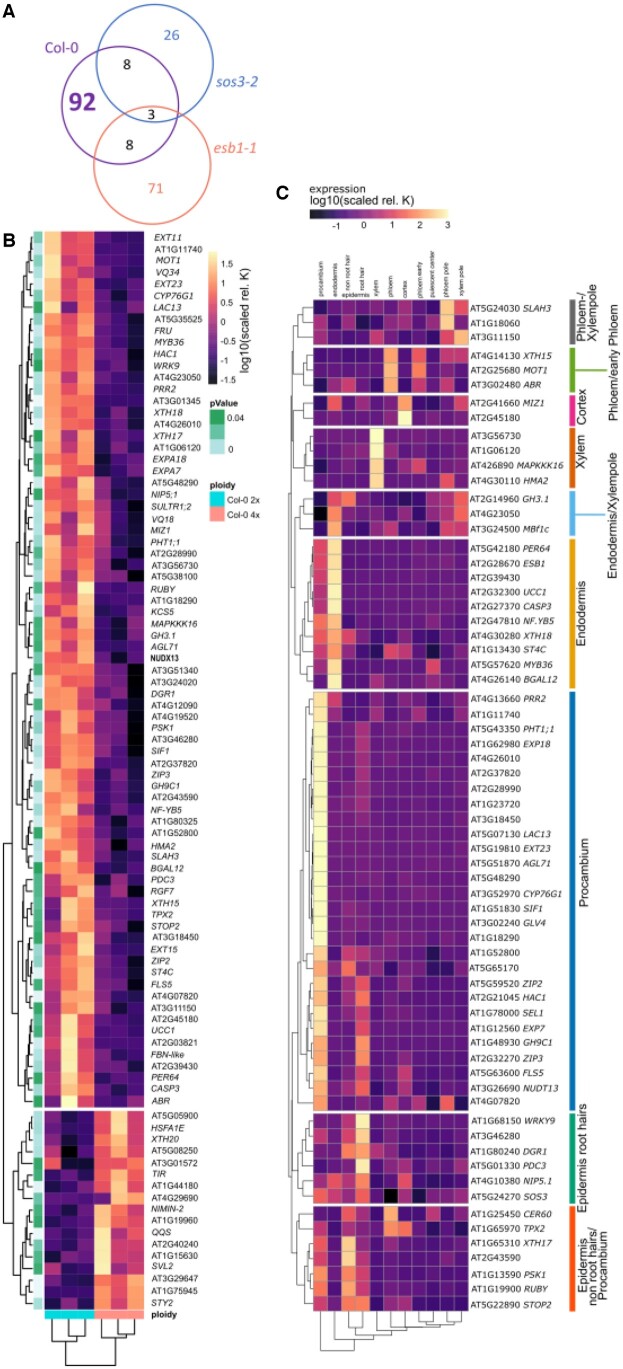
Neo-tetraploid elevated leaf K gene network. A, Venn comparison of all ploidy dependent DEGs reveals that 92 are specifically changed in wild-type but not in the K-phenotype suppressing mutants. B, Heatmap of the expression of 92 genes in diploid and neo-tetraploid wild-type shows a clear ploidy pattern with 17 genes induced in neo-tetraploids and 75 genes repressed. The annotation row indicates significance of fold change of expression between diploid and neo-tetraploid wild-type. Dark: less significant, light: highly significant. C, ePlant http://bar.utoronto.ca/eplant/ was used to obtain data on root cell type-specific expression patterns for 78 selected genes. Data for 66 genes were available on the platform. Expression was normalized to “ROOT_CTRL” and averaged within procambium, endodermis, epidermis nonroot hair, epidermis root hair, xylem, cortex, early phloem, phloem, quiescent center, phloem pole, and xylem pole. A heatmap of the normalized average expression was generated and clustered. High expression is indicated by bright colors, low expression by dark colors. Clusters were used to annotate network analysis in Figure 4 using the color scheme indicated next to the heatmap.

From our first RNA-Seq experiment, we generated a list of DEGs with altered expression between roots of wild-type neo-tetraploid and progenitor diploid. This list of DEGs represents genes potentially involved in all the various phenotypes associated with neo-tetraploids. To refine this list of DEGs to just those genes associated with the elevated leaf K of neo-tetraploids compared to progenitor diploids, we performed a second RNA-Seq experiment using roots of wild-type, and the mutants *sos3-2* and *esb1-1* we have identified to suppress elevated leaf K in neo-tetraploids compared to diploid progenitor. Our logic being that DEGs involved in elevated leaf K in wild-type neo-tetraploids should not be differentially expressed between neo-tetraploid and diploid in mutants that suppress the elevated leaf K phenotype of neo-tetraploids. DEGs between wild-type neo-tetraploids and diploids would be classified as being associated with elevated leaf K in neo-tetraploids if they are not differentially expressed between neo-tetraploid and diploid in both of the mutants *sos3-2* and *esb1-1* that suppress the elevated leaf K of neo-tetraploids compared to progenitor diploids. We grew diploid and neo-tetraploid *sos3-2* and *esb1-1* mutants and wild-type in soil, and measured the leaf ionome. Using a two-way ANOVA, we confirmed the elevated leaf K phenotype of wild-type neo-tetraploids compared to progenitor diploid, and the quantitative suppression of this difference in leaf K between neo-tetraploid and progenitor diploid in the two mutants *esb1-1* and *sos3-2* (ploidy [4x] * Gene [*SOS3*] *P* = 0.042, ploidy [4x] * Gene [*ESB1*] *P* = 0.035, Supplemental Figure S5). We used these plants to perform RNA-Seq, and identified DEGs in roots between neo-tetraploids and their diploid progenitors. In wild-type, we identified 114 DEGs, and this number was reduced in both mutants suppressing the elevated leaf K of neo-tetraploids compared to progenitor diploids (Supplemental Figure S6a). We next compared all DEGs between diploids and neo-tetraploids across wild-type, *sos3-2*, and *esb1-1*. This comparison is illustrated in a Venn diagram showing the overlap in DEGs between all three genotypes (Figure 4A). Through this comparison, we identified 92 of the DEGs between wild-type neo-tetraploids and diploid progenitors that are no longer differentially expressed between neo-tetraploids and diploids in the *sos3-2* and *esb1-1* mutants that suppress the difference in leaf K between neo-tetraploids and progenitor diploids. We conclude that the differential expression of these 92 genes between wild-type neo-tetraploids and progenitor diploids is specifically associated with the elevated leaf K we observe in wild-type neo-tetraploids compared to diploid progenitor. A heat map of the expression of this core set of 92 genes in wild-type identified two major clusters, one contains 17 genes that are upregulated after whole-genome duplication, and the other 75 genes that are downregulated after whole-genome duplication (Figure 3B). To learn more about the pathways affected by whole-genome duplication, we performed a GO enrichment analysis, which revealed enrichment of genes involved in xyloglucan processes (cell wall), ion transport (zinc and inorganic anions), and the Casparian strip (Supplemental Figure S6b).

To further assess the function of these genes in the elevated leaf K in neo-tetraploid compared to progenitor diploid, we investigated the cell type-specific expression patterns of this core gene set in the root. We utilized a publicly available database (ePlant, Waese et al., 2017) to search for the cell type-specific expression pattern of all 75 genes downregulated after whole-genome duplication in wild-type neo-tetraploid roots. We focused on genes that are repressed because we reasoned that this repression must have occurred in the cell type the gene is usually expressed in. For genes that are induced, we cannot make any assumptions about where they may be induced in neo-tetraploids, and, therefore, they were excluded from this analysis. We also included the genes we have already established as being necessary for elevated leaf K in neo-tetraploids compared to diploid progenitor, which are *SOS3*, *MBF1c*, and *ESB1.* Root cell type-specific data were available for 66 of the 78 genes. We generated a heat map based on the tissue-specific expression pattern of the selected genes and grouped them into nine clusters. Each cluster defined a pattern of cell type-specific gene expression within the root (Figure 4c). We detected a cluster of 10 genes, repressed in neo-tetraploids, which are mainly expressed in the endodermis. In this cluster, we find *ESB1* which we had already established as being necessary for the ploidy K phenotype. This cluster contains several genes known to be involved in Casparian strip formation, including *ESB1*, *MYB36* the master transcriptional regulator of Casparian strip biogenesis, and *PER64*, *UCC1*, and *CASP3* regulated by *MYB36* (Roppolo et al., 2011; Lee et al., 2013; Kamiya et al., 2015; Reyt et al., 2020). Very closely clustered is one more group of three genes. Here, genes are additionally expressed in the xylem pole. For these, a direct involvement in Casparian strip formation has not been shown. It includes *GH3.1* which is involved in auxin signaling (Di et al., 2021). Additionally, it contains *MBF1c* which we have confirmed to be necessary for the difference in leaf K between neo-tetraploids and progenitor diploid (Figure 2B). Two further clusters encompass genes expressed in the epidermis, some of them in root hair cells, and others in nonroot hair cells. Notable, here is the *WRKY9* transcription factor which has recently been shown to be induced by salt treatment, and to positively regulate *CYP94B3* and *CYP86B1*, leading to increased root suberin and salt tolerance (Krishnamurthy et al., 2021). A large cluster of genes is expressed in the procambium, among them *LAC13* which has recently been shown to also localize to the Casparian strip region (Rojas-Murcia et al., 2020). Several genes in the procambium cluster are also expressed in the epidermis. For example, *EXP7/Expα-1.26*, codes for an expansin. Expansins are a group of proteins involved in cell wall loosening and turgor-driven cell wall expansion (Li et al., 2002). Expression levels of *EXP7* are positively related with root hair length (Lin et al., 2011). The last clusters encompass stele and cortex-specific genes. Changes in expression in *MAPK^3^16*, which is downregulated in neo-tetraploids, may alter a signaling pathway which could in turn affect K^+^ translocation to the shoot. MAPK^3^16 is expressed in the xylem (Figure 4C). This analysis allowed us to conclude which root tissues are responsive to whole-genome duplication and especially highlighted the importance of the endodermis, already highlighted in the GO enrichment analysis (Supplemental Figure S6b). In some cases, like *MYB36*, a regulator of the DEGs *PER64*, *UCC1*, and *CASP3*, we also showed that several genes are linked through the same expression network. Whole-genome duplication-dependent changes in signaling likely lie upstream of *MYB36*, and lead to a whole series of gene expression changes. We hypothesize that whole-genome duplication affects a number of processes, such as the formation of the endodermis controlled by regulators such as *MYB36*, through a so far unknown signaling cascade. We postulate that by identifying the regulators for each of the molecular structures or processes changed by whole-genome duplication, such as the endodermis, we can resolve the molecular “ploidy signal” initiating phenotypic changes after whole-genome duplication. We should subsequently be able to modify the expression of these regulators to re-create neo-tetraploid phenotypes in a diploid background. This would allow us to emulate desired traits of neo-tetraploids without the downsides of whole-genome duplication such as reduced fitness (Chao et al., 2013).

**Figure 5:**
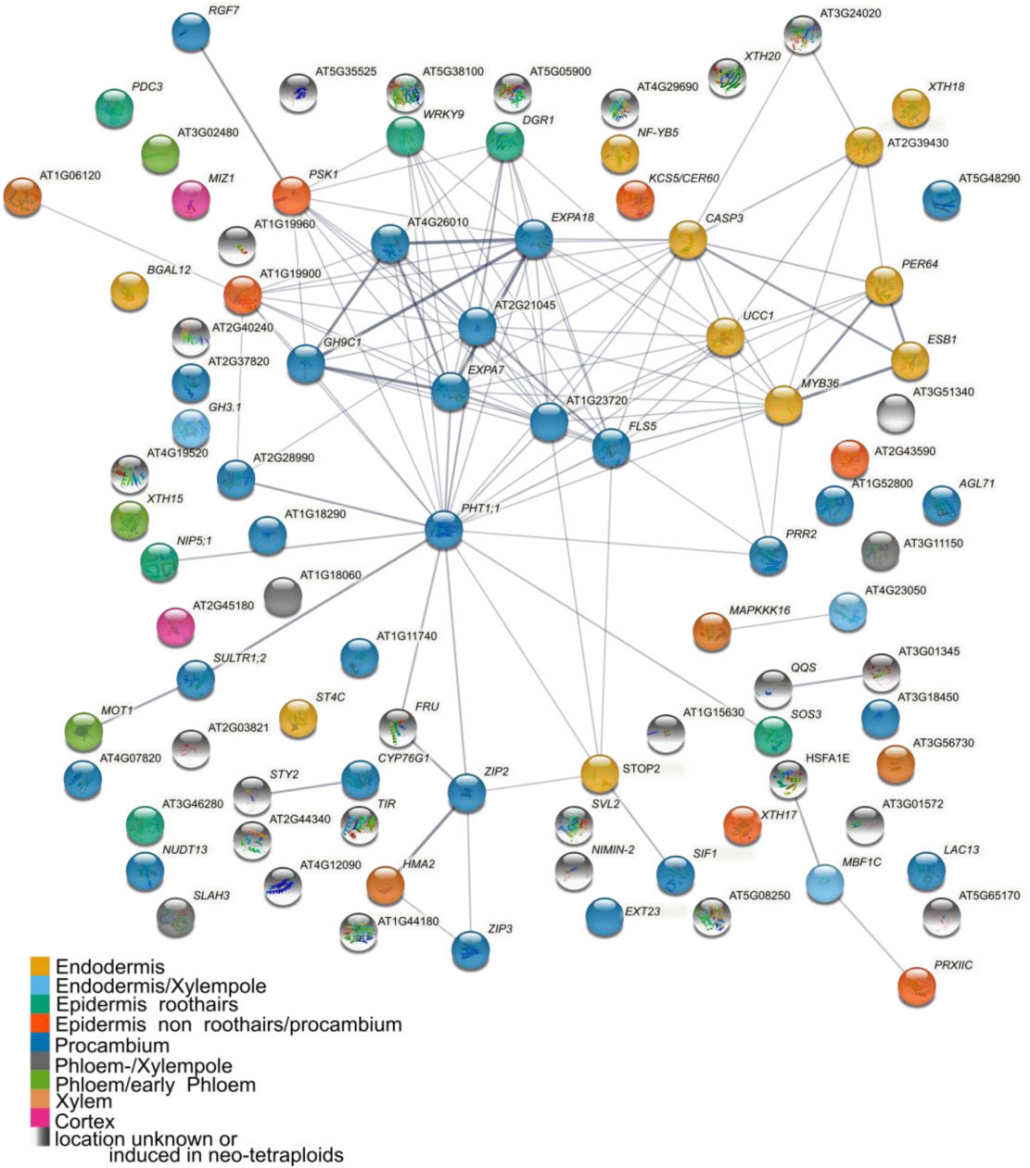
STRING analysis and cell type-specific ploidy changes reveal the importance of the endodermis for increased leaf K concentrations in neo-tetraploids. STING network analysis of 75 downregulated genes plus 3 previously identified genes known to be related to the elevated leaf K phenotype of neo-tetraploids. The STRING network identified hub genes, connected through co-expression correlations with several other genes of the ploidy network. Colors are chosen based on root cell type-specific expression as shown in the public database ePlant. For genes without color no data were available on ePlant or genes were induced after whole-genome duplication.

We performed a network analysis in order to help us identify potential ploidy master regulator genes. Using STRING (Szklarczyk et al., 2015), we assessed connections between the core 92 genes differentially expressed in roots between wild-type neo-tetraploid and progenitor diploid, and determined to be associated with elevated leaf K in the neo-tetraploids. We also included the three genes *ESB1*, *SOS3*, and *MBF1c* we have established genetically to be necessary for the elevated leaf K in neo-tetraploids compared to progenitor diploid (Figure 2B). In this STRING network (Figure 5), genes are connected based on their co-expression correlation. We used the cell type location of expression (Figure 4C) to assign colors to genes in the network. This allowed us to show not only co-expression but also co-localization-dependent connections between genes.

We detect several hub genes; genes co-expressed with numerous other genes. One of these hubs is *EXPA7*, expressed in the procambium and in root hair epidermal cells. *EXPA7* is connected to several endodermal genes, and other root hair and nonroot hair genes. Among them is *PHT1;1*, which encodes for one of two major phosphate uptake transporter in roots, the other one being *PHT1;4*. Both play a role in Pi acquisition from low and high Pi environments, and a lack of both genes leads to increased root hair length (Shin et al., 2004). *PHT1;1* is very highly expressed in root hair cells (Mudge et al., 2002) which are important for Pi uptake. This shows that the most prominent cluster is centered around root hairs and cell growth, suggesting a link between root hair development and the establishment of the elevated leaf K phenotype in neo-tetraploids. Root hairs play an important role in K^+^-uptake in diploid Arabidopsis (Ahn et al., 2004). Further, neo-tetraploid Arabidopsis have longer and denser root hairs (Stetter et al., 2015), a phenotype possibly linked to larger cells of neo-tetraploids (Supplemental Figure S7). However, neo-tetraploids of root hair mutants tested in this study still have elevated leaf K compared to the diploid mutant progenitor (Figure 3A). This suggests that alterations in root hairs in neo-tetraploids are a consequence of the mechanisms driving elevated leaf K in neo-tetraploids, but they are not the primary cause. Another hub gene in the network is *MYB36* which is connected to, as expected, many Casparian strip genes, but also several genes expressed in the procambium. This network (Figure 5) represents gene expression changes in roots of neo-tetraploids induced by whole-genome duplication and that are associated with the elevated leaf K of neo-tetraploids. A subset of these genes will be necessary for the elevated leaf K in neo-tetraploids compares to diploid progenitors, while others are likely to be changing as a consequence of expression changes in these causal genes. This network analysis has allowed us to highlight several genes that could function as hubs or “ploidy master regulators” based not only on their co-expression but also localization of expression. Further studies should focus on these genes when investigation the molecular basis for the leaf K phenotype of neo-tetraploids.

### Cell type specificity of whole-genome duplication drives increased leaf K in neo-tetraploids

**Figure 6:**
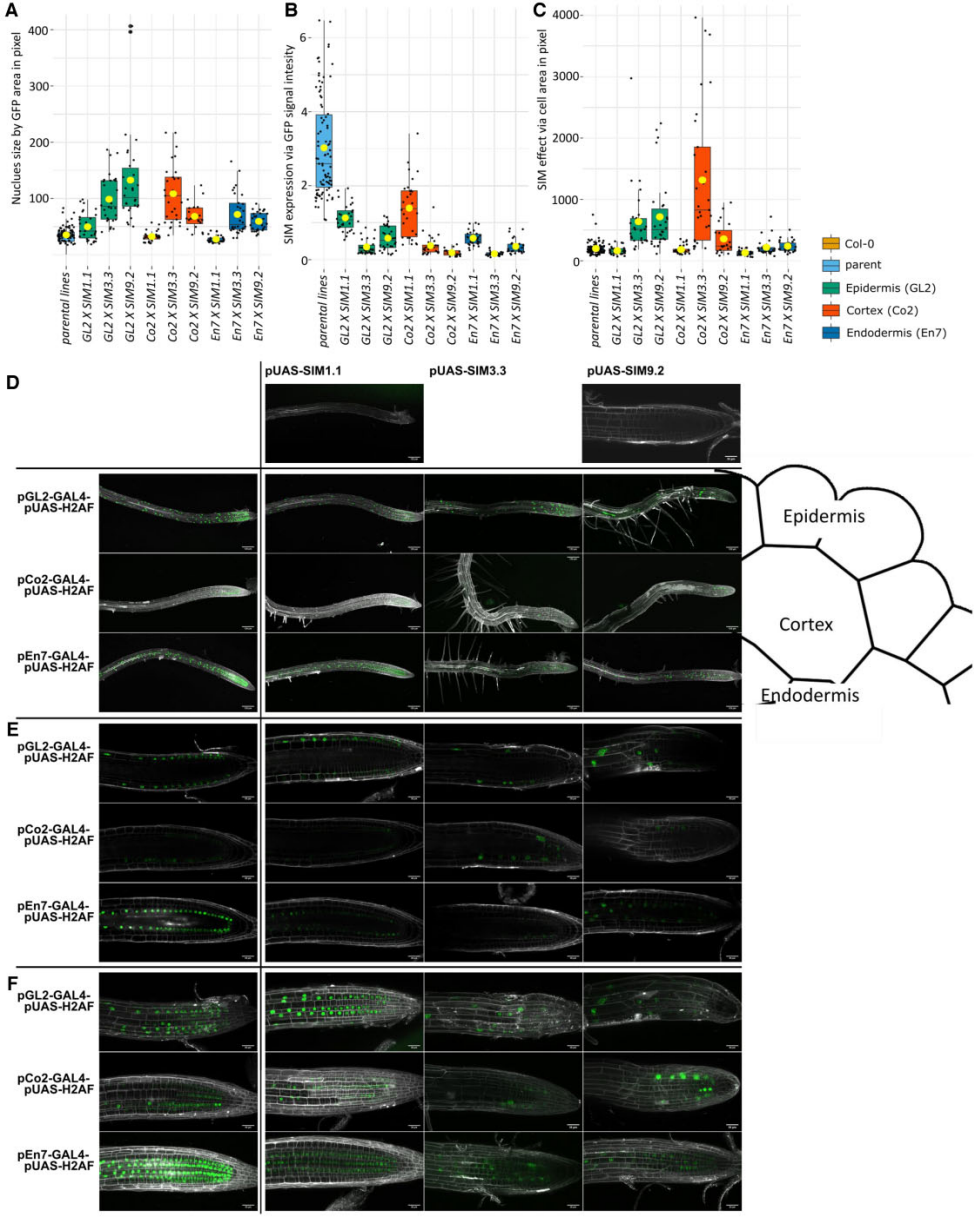
Characterization of SIM overexpression. Microscope images of roots of plants expressing GFP and (in F1 plants of crosses with *pUAS-SIM* lines, parental lines: (*En7*, *GL2*, *Co2*, *SIM1.1*, *SIM3.3*, and *SIM9.2*) SIM in epidermis, cortex, and endodermis. Five-day-old plants, grown on 1/2 MS agar-solidified plates containing no sucrose, are stained with propidium iodide for 3 min. A, Quantification of the GFP signal allows an estimation of *SIM* expression which is higher in the line *En7 X SIM9.2* than *En7 X SIM3.3* and also in *GL2 X SIM9.2* than *GL3 X SIM3.3.* The signal intensity was adjusted to compensate for different gain. B, The effect of SIM on endoreduplication was assessed by measuring the GFP area which is equivalent to the nuclei area. Nuclei of *GL2/Co2/En7 X 1.1* lines are smaller than those of *GL2/Co2/En7 X 9.2* and *3.3* lines, indicating that *SIM* was silenced in this cross, which serves as an additional control. C, The cell area in GFP expressing cells was measured. To avoid the impact of cell elongation cells of the root tips were chosen for this measurement. The effect of *SIM* is related to cell size as expected. *n* = 10–30, One-way ANOVA for A to C showed significant differences between SIM lines and parental lines (Supplemental Table S4). D, Lower magnification shows the early development of root hairs in *GL2/Co2/En7 X SIM* lines. Images are maximum projections GFP gain varied in order to ensure visibility but avoid overexposure. Schematic indicates in which cell-type *SIM* is expressed. Scale bar 120 µm. E, Central region of the root at a higher magnification shows the localization of the GFP signal in the respective tissue. F, Maximum projection through the whole root shows size of nuclei in *GL2/Co2/En7 X 9.2* and *3.3* lines. E and F, scale bar 30 µm. A–C, The center line shows the median, box limits represent the first and third quartiles (the 25th and 75th percentiles), upper and lower whiskers extend to larges or smallest value no further than 1.5 interquartile range and dots beyond whiskers show outliers. Individual values are plotted as dots.

**Figure 7:**
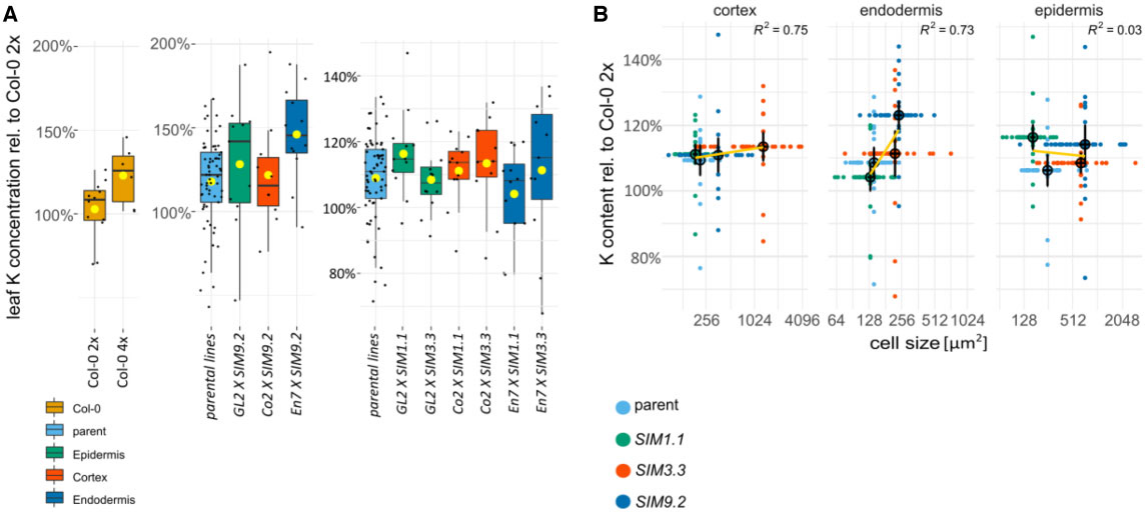
Leaf K in and cell size in lines expressing SIM in tissue-specific patterns. A, Boxplot shows the leaf K concentration relative to diploid wild-type of lines with increase ploidy in epidermis (GL2), cortex (Co2), or endodermis (En7) only (Dietrich et al., 2017). The center line shows the median, box limits represent the first and third quartiles (the 25th and 75th percentiles), upper and lower whiskers extend to larges or smallest value no further than 1.5 interquartile range and dots beyond whiskers show outliers. Individual values are plotted as dots. Ploidy increase was achieved by expression of the endoreduplication gene SIM under control of a tissue-specific promotor. Crosses containing both the promotor and SIM construct display increased endoreduplication, that is polyploidy in one of the three cell types. *n* = 6–65, Diploid (2x), neo-tetraploid (4x). One-way ANOVA showed significant differences between diploids and tetraploids only for the wild-type (Supplemental Table S1). Further ANOVA (Supplemental Table S1) showed significant differences between the parental lines (En7, GL2, Co2, SIM1.1, SIM3.3, and SIM9.2) and En7 X SIM9.2 lines. Lines with a reduced SIM expression (SIM3.3 and SIM1.1) did not show significant differences to parental lines (Supplemental Table S1). As a control we also confirm the known leaf K concentration increase in neo-tetraploid wild-type plants (ANOVA Supplemental Table S1). Yellow dot: averages. Diploid (2x), neo-tetraploid (4x) B, Confocal images (Figure 6) of 7-day-old plate grown (1/2 MS, 1% Agar, no sucrose) were used to measure the root cell size of GFP-positive cells (i.e. cortex, endodermis or epidermis cells, parental lines: (*En7*, *GL2*, and *Co2*) in micrometer square. Average cell size and shoot K concentrations were plotted (black dots: mean ± se) and a linear model, *y–x*, was fitted to measure correlation (yellow line). Individual cell size values are plotted against the average shoot K concentration and individual shoot K concentrations are plotted against the average cell size per genotype to show data distribution. *n*^K concentration^: 10–12; *n*^cell size^: 20–30.

To explore further the set of genes described in the co-expression network (Figure 5), we asked the question, what cell types in the root need to undergo whole-genome duplication to initiate the elevated leaf K observed in neo-tetraploids compared to progenitor diploid? To answer this, we utilized the ectopic expression of *SIM* in a tissue-specific manner. *SIM* is sufficient for endoreduplication (Churchman et al., 2006), the replication of the nuclear genome in the absence of mitosis, which leads to elevated nuclear gene content and polyploidy. Expression of *SIM* in a cell type-specific manner allows targeted endoreduplication in specific cell types (Dietrich et al., 2017). Constitutive overexpression of *SIM* is very disruptive to growth. Previously, a system using *GAL4-VP16*-driven transactivation was established to assess the effect of tissue-specific overexpression in a heterozygous F1 generation (Dietrich et al., 2017). Tissue-specific promotor lines *pEn7-GAL4-pUAS-H2HF, pGo2-GAL4-pUAS-H2AF, pGL2-GAL4-pUAS-H2AF*, and *pUAS* were crossed with *SIM* line *pUAS-SIM9.2* to generate a tissue-specific increase in ploidy. Nuclear localized green fluorescent protein (GFP) was also expressed under control of the tissue-specific promotors, to allow the direct determination of location of SIM expression in the root. Using this approach, we were able to drive endoreduplication specifically in the endodermis (promoter *pEn7*), cortex (promoter *pCo2*), and epidermis (promoter *pGL2*). We assessed nuclei size, GFP signal, and cell size in these F1 plants to directly confirm endoreduplication in the target cell types (Figure 6, A–C). Microscopy of the root showed an early development of long root hairs in lines expressing SIM in the epidermis, cortex, or endodermis (Figure 6D). It also allowed us to confirm the correct tissue localization of the SIM expression (Figure 6, E and F). These plants were grown in soil and the leaf ionome measured. An ANOVA analysis of K in leaves revealed that endoreduplication in the endodermis (strongest effect line *pEn7 X SIM9.2*) produces increased leaf K compared to the parental lines (*P* = 0.003; Figure 7A; Supplemental Table S1). However, we observed no increase in leaf K when endoreduplication is targeted to the cortex (lines *pGL2 x SIM9.2*) (*P* = 0.277, Supplemental Table S1) or epidermis (lines *pCo2 x SIM9.2*) (*P* = 0.695, Supplemental Table S1; Figure 7A). As a control, in the same experiment, we observed an increase in leaf K between wild-type neo-tetraploid and diploid progenitor (*P* = 0.043, Figure 7A; Supplemental Table S1), as expected. Crosses of the cell type-specific promoter lines with two other independent *SIM* lines *SIM1.1* and *SIM3.3*, which have varying levels of SIM activity (Figure 6), allowed us to further evaluate the impact of cell type-specific endoreduplication. We observed the strongest relationship between cell size, as a marker of endoreduplication, and increased leaf K for the endodermis (Figure 7B), with a weaker effect in the cortex, and no effect in the epidermis (Figure 7B). We conclude that whole-genome duplication in the endodermis is sufficient to produce the elevated leaf K observed in wild-type neo-tetraploids compared to diploid progenitor.

## Conclusions

Roots are known to drive the elevated leaf K observed in neo-tetraploids compared to their diploid progenitors. We discovered that disruption of the two major K^+^-uptake systems, AKT1 and HAK5, or their regulators, in roots does not suppress the elevated leaf K in neo-tetraploids caused by whole-genome duplication of the progenitor diploid. We conclude that the elevated leaf K observed in neo-tetraploids compared to diploid progenitor is not due to activation in the neo-tetraploid of the major K^+^-uptake systems in roots. This discovery rules out the most obvious hypothesis regarding the mechanism of elevated leaf K in neo-tetraploids, that it is due to increased activity of AKT1 and/or HAK5 in neo-tetraploids. To explore other possible mechanisms, and build testable hypotheses about this important phenomenon, we tested the potential role of 38 selected genes. We identified three genes (*ESB1*, *SOS3*, and *MBF1c*) for which loss-of-function mutations suppress the elevated leaf K phenotype of neo-tetraploids compared to their diploid progenitors. We show genetically that a functional endodermis, SOS signaling, and certain aspects of upstream ABA signaling are necessary for the elevated leaf K observed in neo-tetraploids compared to their diploid progenitor. Expanding our approach beyond single genes, we uncover a core transcriptional network associated with the increased leaf K concentration in neo-tetraploids. This network contains genes that are either repressed or activated in neo-tetraploid roots. The network is enriched in processes such as root hair development, cell wall re-modeling, ion transport, endodermal development, and ABA signaling. These genes are differentially expressed in key cell types of the root, including procambium, epidermis, root hairs, endodermis, and vascular tissue. Whole-genome duplication appears to initiate root-wide changes in gene expression that lead to elevated leaf K in neo-tetraploids compared to their diploid progenitor. Further, we observe that whole-genome duplication within the endodermis alone is sufficient to drive elevated leaf K concentrations. It remains to be determined genetically which genes within this root-wide core transcriptional network are necessary for elevated leaf K in neo-tetraploids, and which are sufficient. Further, it is an open question as to what the molecular steps initiated by whole-genome duplication in the endodermis are that lead to root-wide transcriptional changes in this core set of genes that drives elevated leaf K in neo-tetraploids. The gene set we have identified provides the basis for the development of testable hypothesis and experimental approaches to help answer these important questions. In certain crops (Meng et al., 2011; An et al., 2014; Wang et al., 2021), tetraploids are more tolerant to drought and salinity. The core gene set we have identified offers the potential to synthetically create, in a diploid background, these beneficial effects of whole-genome duplication without the associated drawbacks, providing an avenue to develop crops more tolerant to drought and salinity.

## Materials and methods

A full description can be found in the Supplemental Materials and Methods, including a list of all primers (Supplemental Table S4) and a full GO enrichment analysis (Supplemental Table S5). Plant lines were obtained from NASC, or donated. A full list can be found in Supplemental Table S6. Elemental content was determined using inductively coupled plasma–mass spectrometry (ICP-MS), image analysis was done using Fiji and statistical analysis and graphs were done using R/RStudio. RNA-Seq was performed by BGI (https://www.bgi.com/global/) according to their standard analysis procedure and by Deep Seq at the University of Nottingham (https://www.nottingham.ac.uk/deepseq/).

## Availability statement

RNA-Seq datasets generated during this study can be found at GEO https://www.ncbi.nlm.nih.gov/geo/ under the number GSE180004 for the assessment of plate grown wild-type plants and GSE180818 for the assessment of soil-grown wild-type and mutant plants. The latter dataset contains further samples not described in this study of plate-grown plants and a fourth mutant *rhd6-3/rsl1-1* and a fifth mutant *chx23-4*. Both sets have also been unified under the SuperSeries GSE180819.

### Accession numbers

Sequence data from this article can be found in the GenBank/EMBL data libraries under accession numbers *ESB1* (AT2G28670), *SOS3* (AT5G24270), *AKT1* (AT2G26650), *HAK5* (AT4G13420), *MYB51* (AT1G18570), *RAP2.11* (AT5G19790), *MBF1C* (AT3G24500), (AT1G06120), (AT1G11740), *EXPA7* (AT1G12560), *ST4C* (AT1G13430), *PSK1* (AT1G13590), (AT1G15630), *FBN-like* (AT1G18060), (AT1G18290), *RUBY* (AT1G19900), (AT1G19960), *EXT15* (AT1G23720), *KCS5* (AT1G25450), (AT1G44180), *GH9C1* (AT1G48930), *SIF1* (AT1G51830), (AT1G52800), *EXPA18* (AT1G62980), *XTH17* (AT1G65310), *TPX2* (AT1G65970), *SVL2* (AT1G66970), *WRKY9* (AT1G68150), *TIR* (AT1G72930), (AT1G75945), *SULTR1;2* (AT1G78000), *DGR1* (AT1G80240), (AT1G80325), *GH3.1* (AT2G14960), *HAC1* (AT2G21045), *MOT1* (AT2G25680), *CASP3* (AT2G27370), *FRU* (AT2G28160), (AT2G28990), *ZIP3* (AT2G32270), *UCC1* (AT2G32300), (AT2G37820), (AT2G39430), (AT2G40240), *MIZ1* (AT2G41660), (AT2G43590), *VQ18* (AT2G44340), (AT2G45180), *NF-YB5* (AT2G47810), (AT3G01345), (AT3G01572), *RGF7* (AT3G02240), *ABR* (AT3G02480), *HSFA1E* (AT3G02990), (AT3G11150), (AT3G18450), (AT3G24020), *NIMIN-2* (AT3G25882), *NUDX13* (AT3G26690), (AT3G29647), *QQS* (AT3G30720), (AT3G46280), (AT3G51340), *CYP76G1* (AT3G52970), (AT3G56730), (AT4G07820), *NIP5;1* (AT4G10380), (AT4G12090), *PRR2* (AT4G13660), *XTH15* (AT4G14130), (AT4G19520), (AT4G23050), (AT4G26010), *BGAL12* (AT4G26140), *MAPKKK16* (AT4G26890), (AT4G29690), *HMA2* (AT4G30110), *XTH18* (AT4G30280), *STY2* (AT4G36260), *PDC3* (AT5G01330), (AT5G05900), *LAC13* (AT5G07130), (AT5G08250), *EXT23* (AT5G19810), *STOP2* (AT5G22890), *SLAH3* (AT5G24030), (AT5G35525), (AT5G38100), *PER64* (AT5G42180), *PHT1;1* (AT5G43350), *XTH20* (AT5G48070), (AT5G48290), *EXT11* (AT5G49080), *MYB36* (AT5G57620), *ZIP2* (AT5G59520), *FLS5* (AT5G63600), *VQ34* (AT5G65170), and *AGL71* (AT5G51870), (AT2G03821).

## Supplemental data

The following materials are available in the online version of this article.

**Supplemental Materials and Methods.**

**Supplemental Figure S1.** Role of ABA-signaling—RNA-Seq of diploid and neo-tetraploid wild-type plants grown on 1/4 Hoagland’s, agar-solidified medium.

**Supplemental Figure S2.** RNA-Seq in diploids and neo-tetraploids reveals K homeostasis genes are differentially regulated.

**Supplemental Figure S3.** Expression of Low K signaling in neo-tetraploids.

**Supplemental Figure S4.** Ploidy-dependent ionomic differences.

**Supplemental Figure S5.** Leaf K for soil-grown plants used for RNA-Seq.

**Supplemental Figure S6.** Neo-tetraploid elevated leaf K gene network.

**Supplemental Figure S7.** Cell size of neo-tetraploids.

**Supplemental Figure S8.** Generation of new tetraploid lines.

**Supplemental Figure S9.** PANTHER GO-enrichment analysis of the effect of Na on the root transcriptome.

**Supplemental Figure S10.** PANTHER GO-enrichment analysis of shoot transcriptome.

**Supplemental Table S1.** ANOVA (Analysis of Variance) for Figures 1, 3, and 4 as well as Supplemental Figures S3, S4, S7, and S10.

**Supplemental Table S2**. Raw ionomic data in parts per million (ppm) or µg/gDW (dry weight).

**Supplemental Table S3.** List of all differentially expressed genes.

**Supplemental Table S4.** List of primers used in this study.

**Supplemental Table S5.** Full results of GO enrichment analysis of a subset of genes DE between wild-type diploid and neo-tetraploids in roots either under control conditions, or under Na stress or both.

**Supplemental Table S6.** List of Arabidopsis lines used and generated for this study.

## Supplementary Material

kiac360_Supplementary_Data

## Notes

S.F. acquired funding, designed and conducted experiments, analyzed results, and wrote the manuscript. P.F. conducted experiments. F.J.Z. contributed to funding and the manuscript, D.E.S. designed experiments, acquired funding, and wrote the manuscript.

The author responsible for distribution of materials integral to the findings presented in this article in accordance with the policy described in the Instructions for Authors (https://academic.oup.com/plphys/pages/general-instructions) is Sina Fischer (sina.fischer@nottingham.ac.uk).
