# Supplementary material for: Transcriptional network underpinning ploidy-related elevated leaf potassium in neo-tetraploids
Source: Plant Physiol. 2022 Aug 5;190(3):1715–30. doi: 10.1093/plphys/kiac360 (PMC9614460; doi:10.1093/plphys/kiac360)
Supplement: kiac360_Supplementary_Data [file kiac360_supplementary_data.zip › PP2022RA00386R1_Supplemental_Material.pdf]

Supplementary Information for

Transcriptional networks underpinning ploidy related increased leaf potassium in neo-tetraploids.

Sina Fischer[1], Paulina Flis[1], Fang-Jie Zhao[2], David E. Salt[1]*
[1]Future Food Beacon of Excellence and the School of Biosciences, University of Nottingham, Nottingham LE12 5RD, UK
[2]State Key Laboratory of Crop Genetics and Germplasm Enhancement, College of Resources and Environmental Sciences, Nanjing Agricultural University, Nanjing, China

*David E. Salt; Email: david.salt@exmail.nottingham.ac.uk

This PDF file includes:

Supplementary text: Materials and Methods (Extended)
Supplemental Figure S1 to S10
Tables S1 to S4
SI References

Supplemental Materials and Methods.

<u>Arabidopsis (*Arabidopsis thaliana*) lines</u>

Arabidopsis lines were obtained from the Nottingham Arabidopsis Stock Center (NASC) and donated by Francisco Rubio (Ragel et al., 2015), Daniela Dietrich (Dietrich et al., 2017), Guilhem Reyt (Roppolo et al., 2011), Priya Ramakrishna (Ramakrishna et al., 2019). The wild type Col-0 neo-tetraploid lines were obtained from Chao *et al*. (Chao et al., 2013). Further, previously published lines were donated by Rahul Bhosale and Malcolm Bennett (Seifert et al., 2002; Foreman et al., 2003; Young et al., 2006; Menand et al., 2007; Yi et al., 2010).

<u>Cultivation conditions</u>

Agar solidified nutrient media: Plants were grown on ½ MS (2.2g MS/l, 1% (w/v) agar, 5mM MES), ¼ or $^{1}/_{10}$ Hoagland medium (250/ 100 µM $NH_4H_2PO_4$, 500/ 200 µM $MgSO_4$, 700/ 280 µM Ca $(NO_3)_2$, 1500/ 600 µM KNO_3, 12.5/ 5 µM Fe-HBED, 5 mM MES at pH 5.7.) solidified with 1 % (w/v) agar (Type A, Sigma Aldrich or Alfa Aesar Low EEO Agarose for high purity agar). In some cases the nutrient solution contained and 1 % (w/v) sucrose (Sigma Aldrich BioXtra). Cultivation was under long day conditions of 16h light (170-230 µmol m^{-2} s^{-1}) /8h dark.

Soil experiments: Plants cultivated on soil was carried out as described previously (Danku et al., 2013; Campos et al., 2021). In brief, plants were grown for 5 weeks on peat Jiffy® soil pellets or in

pots filled with peat substrate (Levington M3, T34 biocontrol®) after stratification for 48h at 4°C. Genotypes were randomized. Plants were bottom watered with nutrient solution (100 µM $NH_4H_2PO_4$, 200 µM $MgSO_4$, 400 µM Ca $(NO_3)_2$, 600 µM KNO_3, 5 µM Fe-HBED, 4.63 µM H_3BO_3, 0.032 µM $CuSO_4$, 0.915 µM $MnCl_2$, 0.077 µM $ZnSO_4$, 0.011 µM MoO_3; pH 5.7 buffered with 5mM MES) once a week. Leaf tissue was harvested for ICP-MS analysis using a scalpel. Leaf samples were washed 3 times in MilliQ H_2O to remove particles adhering to the outside before they were dried at 80°C for 24h. Root tissue was harvested by upending the contents of one pot into a large glass bowl. Roots were scooped from between the loose soil using tweezers and placed into water very briefly. Swirling them in water for a few seconds removed most of the adhering soil. Roots were removed and placed into fresh water containing a few drops of hair conditioner. The mixture was sonicated for 5 seconds and the now clean roots were rinsed in fresh water for a few seconds once more. Finally, roots were frozen in liquid nitrogen. From start to finish the root harvesting was finished within 2 minutes.

Inductively Coupled Plasma-Mass Spectrometry

Sample preparation as described previously (Danku et al., 2013). Dried plant material was digested with 1ml concentrated trace metal grade nitric acid Primar Plus (Fisher Chemicals) spiked with Indium as an internal standard. Samples were heated in dry block heaters (SCP Science; QMX Laboratories) at 115°C for 4h. After cooling, digested samples were diluted to 10ml with 18.2 MΩcm Milli-Q Direct water (Merck Millipore) and subsequently analyzed using an ICP-MS, PerkinElmer NexION 2000 equipped with Elemental Scientific Inc. autosampler, in the collision mode (He). Twenty-four elements were monitored including the following stable isotopes: 7Li, 11B, 23Na, 24Mg, 31P, 34S, 39K, 43Ca, 48Ti, 52Cr, 55Mn, 56Fe, 59Co, 60Ni, 63Cu, 66Zn, 75As, 82Se, 85Rb, 88Sr, 98Mo, 111Cd, 208Pb and 115In. Liquid reference material composed of pooled samples was prepared before the beginning of a sample run and was used throughout the whole sample run. It was run after every ninth sample to correct for variation within ICP-MS analysis run. The calibration standards (with indium internal standard and blanks) were prepared from single element standards (Inorganic Ventures; Essex Scientific Laboratory Supplies Ltd, Essex, UK) solutions. Sample concentrations were calculated using external calibration methods within the instrument software. The final elements concentrations were obtained by normalizing the elements concentrations to the samples dry weight.

Confocal microscopy

Plants were grown on agar solidified ½ MS medium for 5 days, then stained in 6 µg/ml propidium iodide for 2 min and mounted on slides using cover slips No 1.5. A Leica SP5 Confocal microscope was used to image roots. Laser power was set at 3%, collection was between 600-620 nm, smart Gain at 12. Bidirectional scanning with 400Hz scan speed was used to acquire image z stacks. Confocal images were assessed for cell size and assembled using Fiji (Schindelin et al., 2012).

Confirmation of T-DNA insertions

Leaves were used for gDNA extraction. Material was frozen, pulverized and 500 µl extraction buffer (200 mM Tris/HCl pH 7.5, 250 mM NaCl, 25 mM EDTA, 0.5 % (w/v) SDS) were added. DNA contained in the aqueous phase was precipitated using isopropanol (1:1) and washed with ethanol. After resuspension in 50 µl deionized water, gDNA was used for PCR amplification. Two PCR reactions, for wild type and mutant fragment using primers in Table S4, using MyTaq were performed according to the manufacturer's instructions and PCR products separated on a 1% (w/v) Agarose gel in 0.5x TBE buffer.

Analysis of transcript levels

Plant material was frozen in liquid nitrogen and RNA extracted by adding 1 ml of TRIzol® (Invitrogen) to 100 mg of pulverized material. After mixing 200 µl chloroform were added. After renewed shaking, cell debris was separated through centrifugation (12000g, 4°C, 15 min) and RNA in the liquid phase was precipitated by addition of equal volumes of Isopropanol. After centrifugation (12000g, 4°C, 10min) the liquid phase was discarded and the pelleted RNA washed with 70% (v/v) ethanol before solubilizing it in 100 µl RNAse-free water. Further purification was achieved by using the RNAeasy Kit from Qiagen, following the instruction manual of the manufacturer. Purity and concentration were analyzed via Nano-drop. For RN-Seq analysis of RNA from plants grown on agar-solidified ¼ Hoagland grown plants the RNA quality was confirmed via RIN values (Agilent Tapestation System) and subsequently sent for sequencing to BGI Hong Kong. Single end (SE) sequencing was performed on a BGISEQ-500 RS Platform. Library preparation, sequencing, alignment and bioinformatics were performed by BGI according to their standard procedures. In short mRNA was enriched using Oligo (dT) magnetic beads, fragmented and reverse transcribed to double-strand cDNA by N6 random primers. The double stranded cDNA was then end repaired with phosphate at the 5' end and stickiness 'A' at the 3' end, and then

ligated to adaptors with sticky 'T'. Using two specific primers the ligation product was amplified and the PCR product was cyclized by splint oligo and DNA ligases. The sequencing reactions were then performed on the library employing a 50SE strategy. The platform used was a benchtop high throughput sequencer BGISEQ-500 yielding 24,017,754 raw sequencing reads and then 24,007,388 clean reads after filtering low quality. After filtering, clean reads were mapped to reference genome (TAIR10 (Lamesch et al., 2011)) using HISAT and Bowtie2. The average mapping ratio with the reference genome was calculated to be 91.18%. Sequencing saturation and read randomness were checked for each sample before proceeding to ensure good quality. Gene expression as Fragments Per Kilobase of transcript per Million mapped reads (FPKM) was then calculated using the software package RSEM. This quantification tool computes the maximum likelihood abundance estimates by applying the statistical algorithm Expectation Maximization (EM). This includes paird-end (PE), variable-length reads, fragment length distributions and quality scores modelling to determine which transcripts are isoforms of one gene. The formula to calculate FPKM is: $FPKM = \frac{10^6 C}{NL/10^3}$. The benefit of this method is to eliminate the influence of differences in length as well as sequencing differences between samples on the gene expression calculation. Finally, differentially expressed genes were extrapolated by use of the NOISeq method. First, the noise distribution is modelled by calculating the log2 fold changes (M) from each samples gene expression and the absolute different value (D) of all pair conditions: $M^i = log_2\,(\frac{x_1^i}{x_1^i})$ and $D^i = \left|x_1^i - x_2^i\right|$. Then for each gene, the fold change is determined from the average expression in 2 different groups calculated from the three replicates of plants, pooled to gain at least 100mg fresh material, from one experiment, and absolute different values for each gene will be calculated: $M_A = log_2\,(\frac{sample1\ avg}{sample2\ avg})$ and $D_A = |sample1\ avg - sample\ 2\ avg|$. M_A and D_A have to be different from the noise distribution model for the gene to be considered a differentially expressed gene (DEG). DEGs were then filtered for a fold change ≥2 and a diverge probability ≥0.8.

For RNA-Seq analysis of RNA from plants grown on agar solidifed ½ MS or soil RNA was isolated using the protocol from (Paredes et al., 2018). RNA was send for sequencing at Deep Seq University of Nottingham. In brief, RNA concentrations were measured using the Qubit Fluorometer and the Qubit RNA BR Assay Kit (ThermoFisher Scientific; Q10211) and RNA integrity was assessed using the Agilent TapeStation 4200 and the Agilent RNA ScreenTape Assay Kit

(Agilent; 5067-5576 and 5067-5577). For each sample cDNA was generated from 200ng of total RNA using the QuantSeq 3' mRNA-Seq library prep kit (Lexogen; 5001-5004). Indexed sequencing libraries were prepared using the Lexogen i7 6nt Index Set (Lexogen; 7001-7096). Libraries were quantified using the Qubit Fluorometer and the Qubit dsDNA HS Kit (ThermoFisher Scientific; Q32854). Library fragment-length distributions were analyzed using the Agilent TapeStation 4200 and the Agilent High Sensitivity D1000 ScreenTape Assay (Agilent; 5067-5584 and 5067-5585). Libraries were pooled in equimolar amounts and final library quantification performed using the KAPA Library Quantification Kit for Illumina (Roche; KK4824). The library pool was sequenced on the Illumina NextSeq 500 using a NextSeq 500 High Output Kit v2.5 75 cycle kit (Illumina; 20024906), to generate approximately 5 million 75bp single-end reads per sample. The raw data were imported into Galaxy Europe (Afgan et al., 2016) and Salmon was used to quantify reads (Patro et al., 2017) and 3D RNA-Seq (Guo et al., 2020) used to filter, normalize and qc data, and to calculate DEGs. The package is able to calculate splice variants but only differential expression was used for this analysis since libraries were prepared such that only the 3' end was transcribed. Read counts and transcript per million reads (TPMs) were generated using tximport R package version 1.10.0 and scaledTPM method (Soneson et al., 2016) with inputs of transcript quantifications from Salmon (Patro et al., 2017). Low expressed genes were filtered based on analysing the data mean-variance trend. The expected decreasing trend between data mean and variance was observed when expressed transcripts were determined to have ≥ 6 of the 90 samples with count per million reads (CPM) ≥ 2, which provided an optimal filter of low expression. A gene was expressed if any of its transcripts with the above criteria was expressed. The TMM method was used to normalize the gene and transcript read counts to log_2-CPM (Bullard et al., 2010). A principal component analysis (PCA) plot showed the RNA-seq data did not have distinct batch effects. For differentially expressed genes, the log_2 fold change (L_2 FC) of gene/transcript abundance were calculated based on contrast groups and significance of expression changes were determined using t-test. P-values of multiple testing were adjusted with BH to correct false discovery rate (FDR) (Benjamini and Yekutieli, 2001). A gene was significantly differentially expressed in a contrast group if it had adjusted p-value < 0.05 and L_2 FC ≥ 0.5. Number or replicates for this experiment was 3. Plate grown plants were pooled to gain enough material for RNA extraction while for soil grown plants one plant represents one replicate. Replicates originate from one experiment.

<u>Generation of neo-tetraploid lines</u>

The previously established protocols were modified and combined. Seeds of diploid progenitors were sown on Jiffies® and germinated in long day conditions. 20 seedling per genotype, at the 2-leaf stage were treated with Colchicine (0.25% (w/v) in H_2O). One drop was placed on the shoot meristem. Contrastingly to the original protocol this was carried out on plants germinated on soil instead of plates (Yu et al., 2009). By circumventing transferring plants to soil after their colchicine treatment the stress of the transfer can be avoided (Supplemental Figure S9). Additionally, the workload is reduced. Plants which have been treated with colchicine stall in their development and only after 7 to 10 days it becomes apparent which of the shoot apexes will recover from the treatment. Surviving plants were grown to seed maturity. Seeds were sown and the germinating seedlings were pre-screened for increased trichome branching (Yu et al., 2009) and grown for 5 weeks (Supplemental Figure S9) under short day conditions. At this stage one leaf was harvested and tested for whole genome duplication (WGD) using flow cytometry. The isolation protocol was adapted (Henry et al., 2005; Wu et al., 2009) for high throughput. In this manner the isolation and analysis of 60 samples can be achieved in one day. The leaves were mechanically disrupted by peeling the lower leaf epidermis using tape (Wu et al., 2009). The exposed leaves were shaken in 600µl extraction buffer (15mM HEPES, 1mM Na-EDTA, 80mM KCl, 20mM NaCl, 200mM Sucrose, 0.2% Triton-X (v/v), 0.5mM Spermin, pH 6.1), 0.3µl RNase (100ng/µl) was added and samples were incubated at RT for 10min. 10µl of PI (1mg/ml) was added and samples stored at 4°C, in the dark for at least 1h until analysis. 400µl of each sample were transferred to analysis tubes and run on the DB FACS Canto. Settings for the machine: FSC 376V, SSC 330V, PI 369V, cut-off 35000, events 10 – 50 thousand. On a $logSSC_{area}$ vs $logPI_{area}$ plot distinct fractions were gated and displayed in a PI_{area} histogram. Data were analyzed using Kaluza software (Version 2.1.00001.20653). Ploidy was determined by the highest peak from the histogram, diploid and tetraploid Col-0 nuclei extracts were used to identify 2x and 4x peaks. Plants analyzed via FACS were transferred to long day conditions and further grown until seed maturity. Seeds harvested from these plants were collected for phenotypic analysis of neo-tetraploid mutant lines. Nomenclature: Diploid mutant lines were described as per general conventions ie. *hak5* gene, line *hak5-3* referring to line N574868 from the NASC stock collection or SALK_074868.54.75.x. Individual colchicine treated plants were given consecutive number ie. *hak5-3_1* followed by *hak5-3_2* etc. Lines derived from these independent colchicine treated plants were annotated with #consecutive numbers ie. *hak5-3_1#1* or *hak5-3_2#1* represent two independent duplication events of the same diploid progenitor. Additionally, chromosomal

spreads of floral buds of plants selected via flow cytometry were prepared (Higgins et al., 2014) and chromosomes were counted (Supplemental Figure S9). Neo-tetraploid nuclei contain 20 chromosomes which were visible during the metaphase or first condensation during prophase (Supplemental Figure S9).

Statistical analysis

Figures were created and statistical analyses were performed with R version 4.1.0 (2021-05-18) using RStudio Version 1.0.143 (www.rstudio.com). Platform: x86_64-w64-mingw32/x64 (64-bit), Running under: Windows 10 x64 (build 19043). The following packages were used: PCAtools (version 2.4.0), ggrepel (version 0.9.1), nsprcomp (version 0.5.1-2) (Sigg and Buhmann, 2008), factoextra (version 1.0.7), FactoMineR (version 2.4) (Lê et al., 2008), RFLPtools (version 1.9), clValid (version 0.7), pheatmap (version 1.0.12), heatmaply (version 1.2.1), plotly (version 4.9.4.1), dendextend (version 1.15.1), veccompare (version 0.1.0), VennDiagram (version 1.6.20), futile.logger (version 1.4.3), scales (version 1.1.1), kableExtra (version 1.3.4), knitr (version 1.33), flextable (version 0.6.7), corrplot (version 0.90), Hmisc (version 4.5-0), Formula (version 1.2-4), devtools (version 2.4.2), usethis (version 2.0.1), ggpmisc (version 0.4.0), ggpp (version 0.4.1), gplots (version 3.1.1), cowplot (version 1.1.1), sjPlot (version 2.8.9), bestNormalize (version 1.8.0), multcompView (version 0.1-8), multcomp (version 1.4-17), TH.data (version 1.0-10), MASS (version 7.3-54), survival (version 3.2-11), mvtnorm (version 1.1-2), emmeans (version 1.6.2-1 ggpubr (version 0.4.0), cluster (version 2.1.2), viridis (version 0.6.1), viridisLite (version 0.4.0), RColorBrewer (version 1.1-2), gridExtra (version 2.3), tidyquant (version 1.0.3), quantmod (version 0.4.18), TTR (version 0.24.2), PerformanceAnalytics (version 2.0.4), xts (version 0.12.1), zoo (version 1.8-9), lubridate (version 1.7.10), ggfortify (version 0.4.12), rcompanion (version 2.4.1), forcats (version 0.5.1), stringr (version 1.4.0), dplyr (version 1.0.7), purrr (version 0.3.4), readr (version 2.0.0), tidyr (version 1.1.3), tibble (version 3.1.3), ggplot2 (version 3.3.5), tidyverse (version 1.3.1), Rmisc (version 1.5), plyr (version 1.8.6), lattice (version 0.20-44).

Gene ontology (GO) enrichments were calculated using the PANTHER tool (http://www.pantherdb.org/). A statistical overrepresentation test using the Fisher's Exact test and Bonferroni correction for multiple testing was used to calculated p-values. These take the group size for each GO term into account as well as the number of DEGs in total and the number of GO annotatable genes. The resulting value is thus scaled. Gene ontology terms with $p \leq 0.05$ were selected for interpretation. The hierarchy display was used to select the most detailed group

amongst all related GO terms and results displayed for this group only. The full results can be found in Supplemental Table S5. A GO enrichment for other contrast groups besides root expression in diploid vs neo-tetraploid wild type, which is shown in the main text, can be found in Supplemental Figure S9-S10.

A STRING analysis was performed using the tool https://string-db.org/. A search for multiple proteins by identifiers was performed. Settings were set to “full STRING network”, a minimum interaction score of 0.4, line thickness was given as confidence and only the query proteins were shown. Clustering was not enabled.

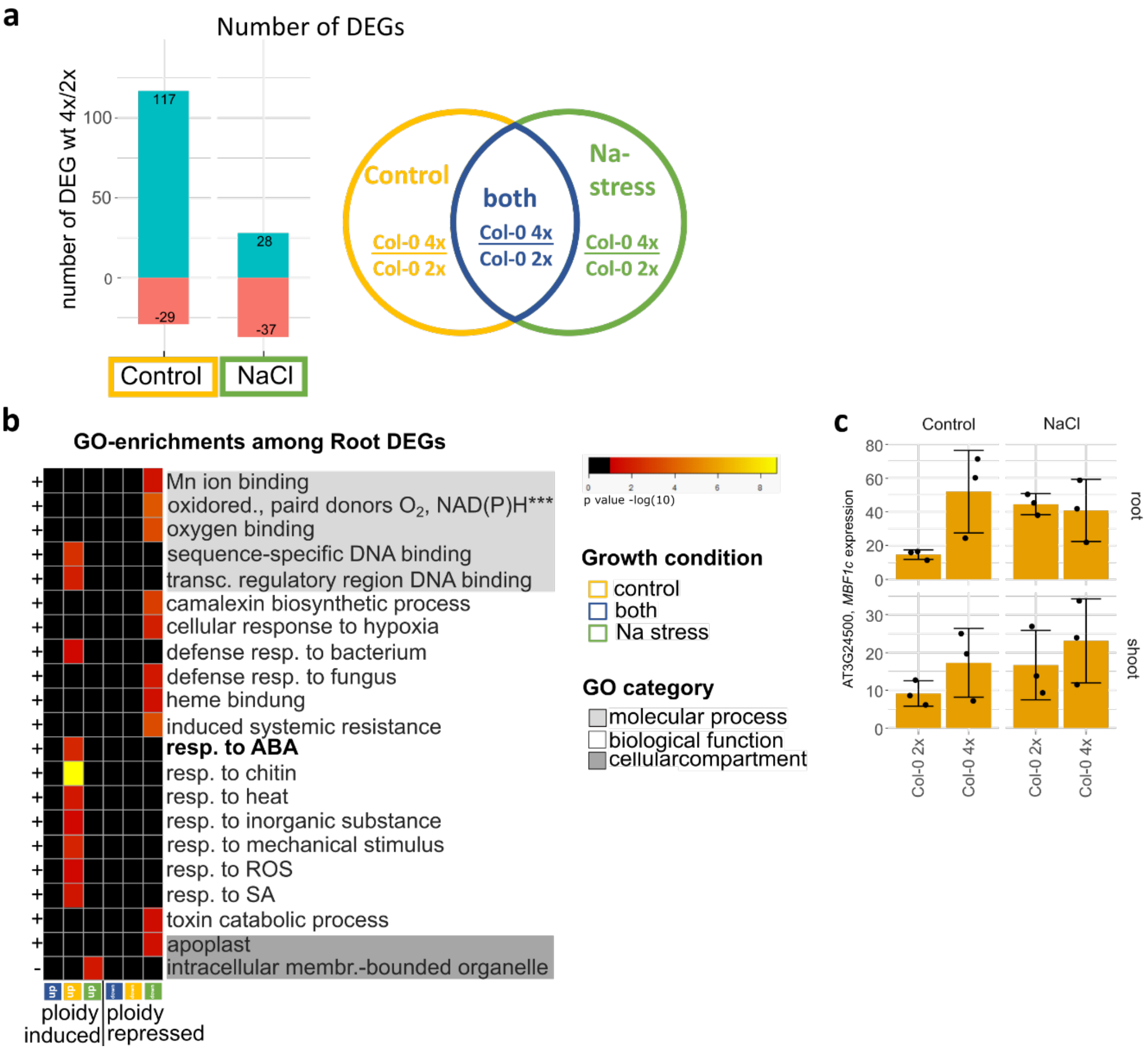

Supplemental Figure S1: Role of ABA-signaling – RNA-Seq of diploid and neo-tetraploid wild-type plants grown on ¼ Hoagland's, agar solidified medium. a) Number of DEGs between diploids and neo-tetraploids (under control and Na stress) are shown for root expressed genes. A Venn diagram shows the comparison between DE genes which was used for the GO analysis in b. Genes were defined as being DE in neo-tetraploids under either control conditions (yellow) or Na stress (green) or both (blue). The color scheme was used in Fig. 5A and S14 to indicate which group of genes was used for the respective GO enrichment analysis. Diploid (2x), neo-tetraploid (4x) **b)** Heatmap shows the p-value of significantly enriched GO terms among genes differentially expressed (DEGs) after WGD in plants grown for two weeks on ¼ Hoagland's, agar solidified media containing sucrose and 0 or 35mM NaCl. For gene selection see Supplementary Fig. 11. Among the enriched classes is "response to ABA" containing 11 genes. It is overrepresented among genes induced in neo-tetraploid roots and is connected to tolerance to abiotic stress. Analysis was done using PANTHER. Displayed are only the most defined terms or child terms. A full list can be found in Supplemental Table S5. Test Type: Fisher's Exact, Bonferroni correction for multiple testing, GO database release 2019-12-09. Abbreviations for GO categories: ***=oxidoreductase activity, acting on paired donors, with incorporation or reduction of molecular oxygen, NAD(P)H as one donor, and incorporation of one atom of oxygen, transc.=transcription, resp.=response, membr.=membrane, ABA = Abscisic acid, ROS= Reactive Oxygen Species, SA= Salicylic acid. **b)** Bar

plots show the RNA-Seq assessed expression (mean Fragments Per Kilobase of transcript per Million mapped reads (FPKM)±SD) of *MBF1c* in plants grown on agar solidified $^{1}/_{4}$ Hoagland with sucrose, with and without salinity stress (35mM NaCl) in root and shoot tissues. n=3, individual samples. Significant increase in expression in roots of control grown plants (cutoff fold change ≥2 and a diverge probability ≥0.8). Diploid (2x), neo-tetraploid (4x)

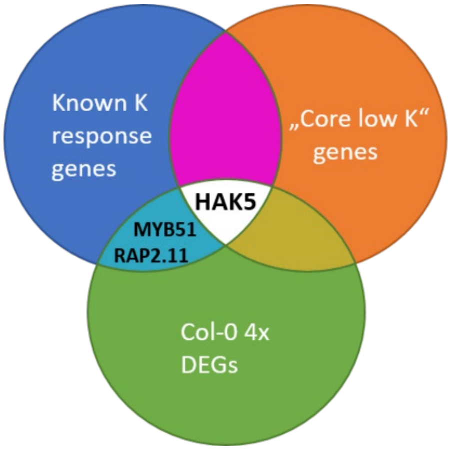

Supplemental Figure S2: RNA-Seq in diploids and neo-tetraploids reveals K homeostasis genes are differentially regulated: The Venn diagram shows the intersections between Differentially Expressed Genes (DEG) after WGD, low K treatment and known *HAK5* regulators. RNA-Seq analysis showed the enhanced expression of *HAK5* in neo-tetraploids which is also among the "Core K deficiency" response genes. In orange: "Core-low K": 20 genes defined by meta analysis of expression studies (Hampton et al., 2004; Gierth et al., 2005; Forieri et al., 2016); In blue: 14 genes, known components of the low K signalling (Hong et al., 2013; Nieves-Cordones et al., 2014); In green: 211 genes differentially expressed in wild-type neo-tetraploid roots.

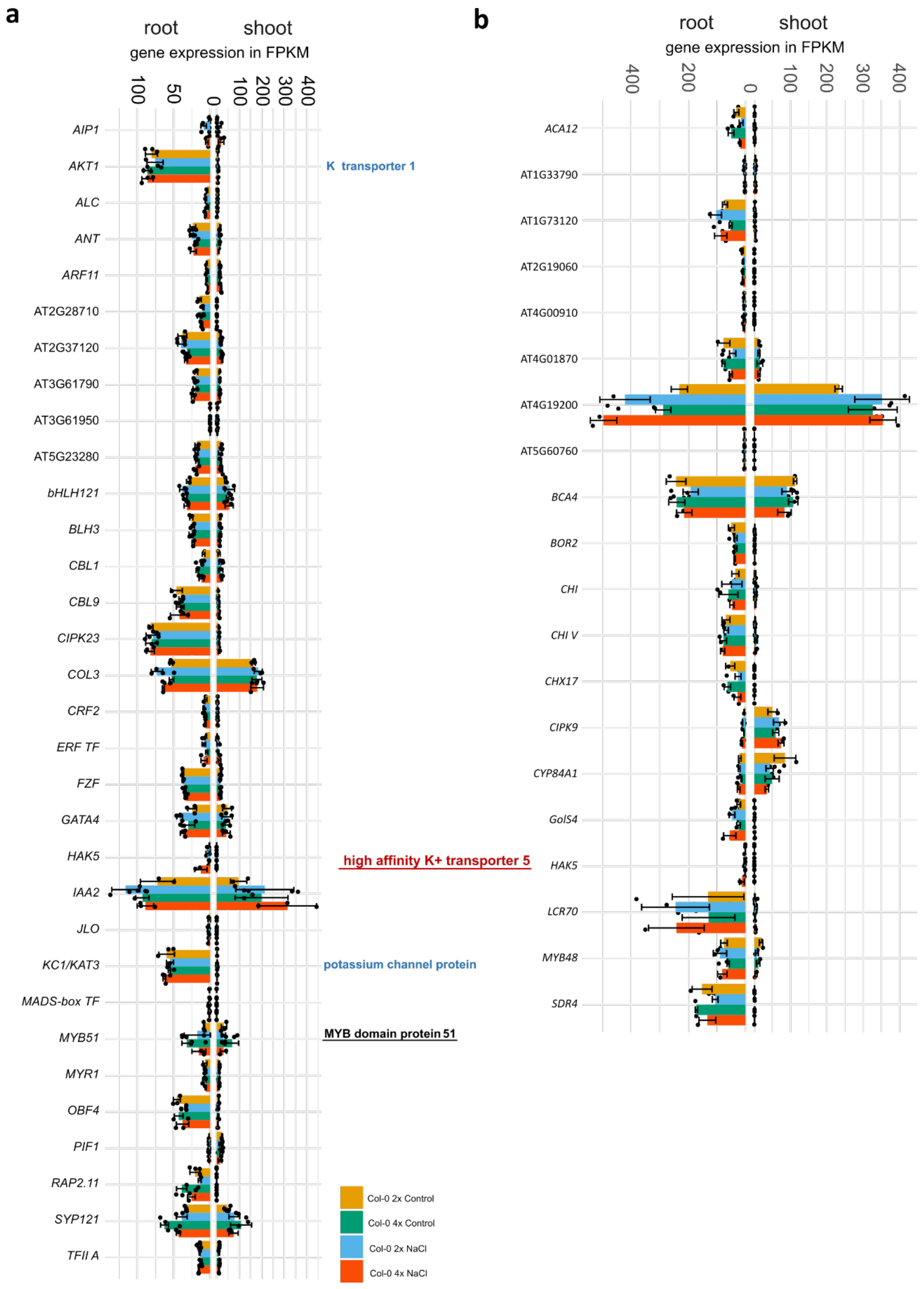
a
root
shoot
gene expression in FPKM
100
50
0
100
200
300
400
AIP1
AKT1
ALC
ANT
ARF11
AT2G28710
AT2G37120
AT3G61790
AT3G61950
AT5G23280
bHLH121
BLH3
CBL1
CBL9
CIPK23
COL3
CRF2
ERF TF
FZF
GATA4
HAK5
IAA2
JLO
KC1/KAT3
MADS-box TF
MYB51
MYR1
OBF4
PIF1
RAP2.11
SYP121
TFII A
K transporter 1
high affinity K+ transporter 5
potassium channel protein
MYB domain protein 51
Col-0 2x Control
Col-0 4x Control
Col-0 2x NaCl
Col-0 4x NaCl
b
root
shoot
gene expression in FPKM
400
200
0
100
200
300
400
ACA12
AT1G33790
AT1G73120
AT2G19060
AT4G00910
AT4G01870
AT4G19200
AT5G60760
BCA4
BOR2
CHI
CHI V
CHX17
CIPK9
CYP84A1
GolS4
HAK5
LCR70
MYB48
SDR4

Supplemental Figure S3: Expression of Low K signalling in neo-tetraploids. The expression of components of the low K signalling machinery as defined by **a)** Nieves-Cordones *et al*., 2014 and Hong et al., 2013 (Hong et al., 2013; Nieves-Cordones et al., 2014) and by **b)** a cross comparison of K starvation genes identified in a gene expression study by Forieri *et al*., 2016, Gierth *et al*., 2005 and Hampton *et al*., 2004 (Hampton et al., 2004; Gierth et al., 2005; Forieri et al., 2016). Bold gene descriptions mark genes for which an induction by low K has been seen as well as a physical interaction with the promotor of *HAK5*. In red and blue are genes for the high affinity and low affinity K uptake system respectively. Underlined are genes defined as DE between diploids and neo-tetraploids. With * are genes defined as DE after Na-stress. n=3+/-se. Diploid (2x), neo-tetraploid (4x)

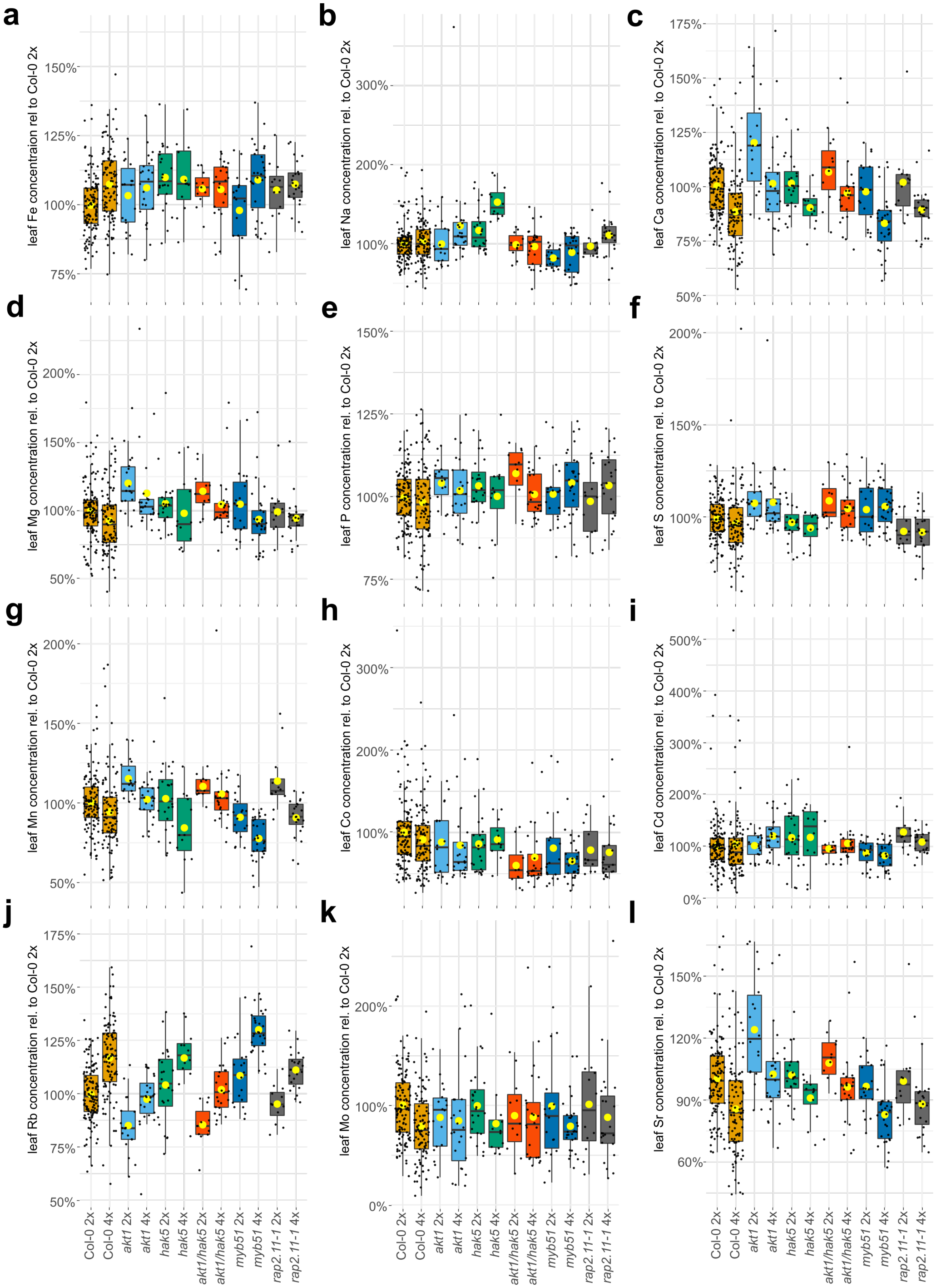
a
leaf Fe concentraion rel to Col-0 2x
150%
125%
100%
75%
b
leaf Na concentration rel. to Col-0 2x
300%
200%
100%
c
leaf Ca concentration rel. to Col-0 2x
175%
150%
125%
100%
75%
50%
d
leaf Mg concentration rel. to Col-0 2x
200%
150%
100%
50%
e
leaf P concentration rel. to Col-0 2x
150%
125%
100%
75%
f
leaf S concentration rel. to Col-0 2x
200%
150%
100%
g
leaf Mn concentration rel. to Col-0 2x
200%
150%
100%
50%
h
leaf Co concentration rel. to Col-0 2x
300%
200%
100%
i
leaf Cd concentration rel. to Col-0 2x
500%
400%
300%
200%
100%
0%
j
leaf Rb concentration rel. to Col-0 2x
175%
150%
125%
100%
75%
50%
k
leaf Mo concentration rel. to Col-0 2x
200%
100%
0%
l
leaf Sr concentration rel. to Col-0 2x
150%
120%
90%
60%
Col-0 2x
Col-0 4x
akt1 2x
akt1 4x
hak5 2x
hak5 4x
akt1/hak5 2x
akt1/hak5 4x
myb51 2x
myb51 4x
rap2.11-1 2x
rap2.11-1 4x

m

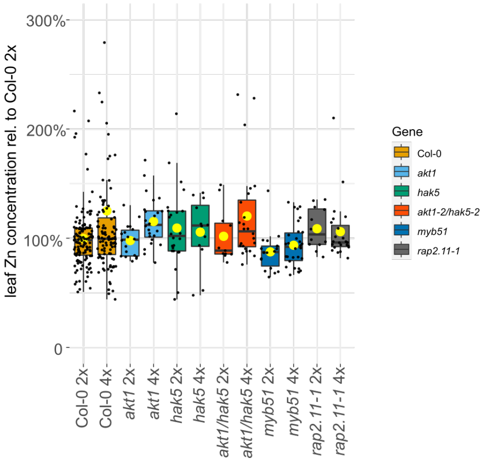

Supplemental Figure S4: Ploidy-dependent ionomic differences. **a-n)** The leaf ionome of diploid and neo-tetraploid Arabidopsis wild-type (Col-0) plants was assessed. The results show genotype dependent changes. The center line shows the median, box limits represent the first and third quartiles (the 25th and 75th percentiles), upper and lower whiskers extend to larges or smallest value no further than 1.5 inter-quartile range and dots beyond whiskers show outliers. Individual values are plotted as dots. Plants were grown on peat-based soil for 5 weeks. n= 11-121, The two way ANOVA (Sup Table 4) showed significant differences between diploids and neo-tetraploids on Rb concentration. Yellow dot: averages. All elements within the limit of quantification (LOQ) were plotted. Diploid (2x), neo-tetraploid (4x)

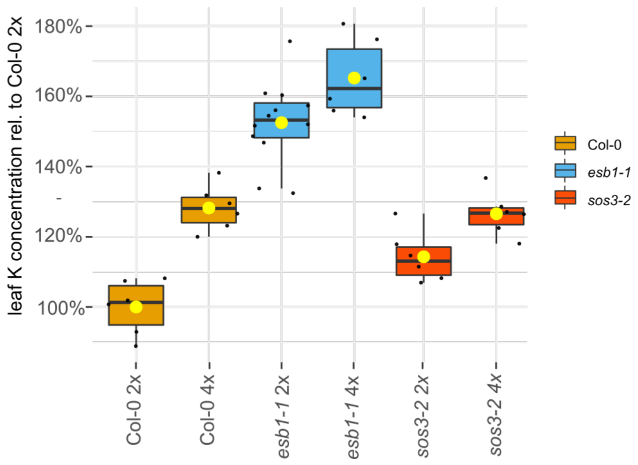

Supplemental Figure S5: Leaf K for soil-grown plants used for RNA-Seq. Boxplots show the K concentration relative to the leaf K content of diploid wild type. The center line shows the median, box limits represent the first and third quartiles (the 25th and 75th percentiles), upper and lower whiskers extend to larges or smallest value no further than 1.5 inter-quartile range and dots beyond whiskers show outliers. Individual values are plotted as dots. n= 6. A two way ANOVA revealed significant differences in leaf K concentration by ploidy, and a significant interaction between gene * ploidy (Sup Table 4).

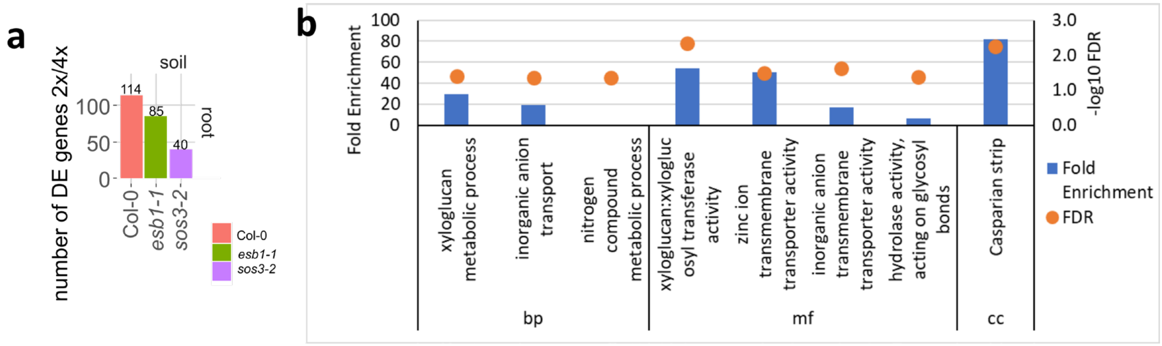

Supplemental Figure S6: Neo-tetraploid elevated leaf K gene network. a) Number of ploidy related gene expression changes (differentially expressed (DE) genes) in roots of soil grown plants shows fewer differences in neo-tetraploid mutants suppressing the elevated leaf K phenotype of neo-tetraploids. **b)** Graph displaying fold enrichment and significance (-log10 false discovery rate FDR) of GO categories among the 92 genes. Analysis was done using PANTHER. Displayed are only the most defined terms or child terms. A full list can be found in Supplemental Table S5. Test Type: Fisher's Exact, Bonferroni correction for multiple testing, GO database release 2019-12-09. Abbreviations: bp= biological process; mf= molecular function; cc= cellular compartment; FDR= False discovery rate

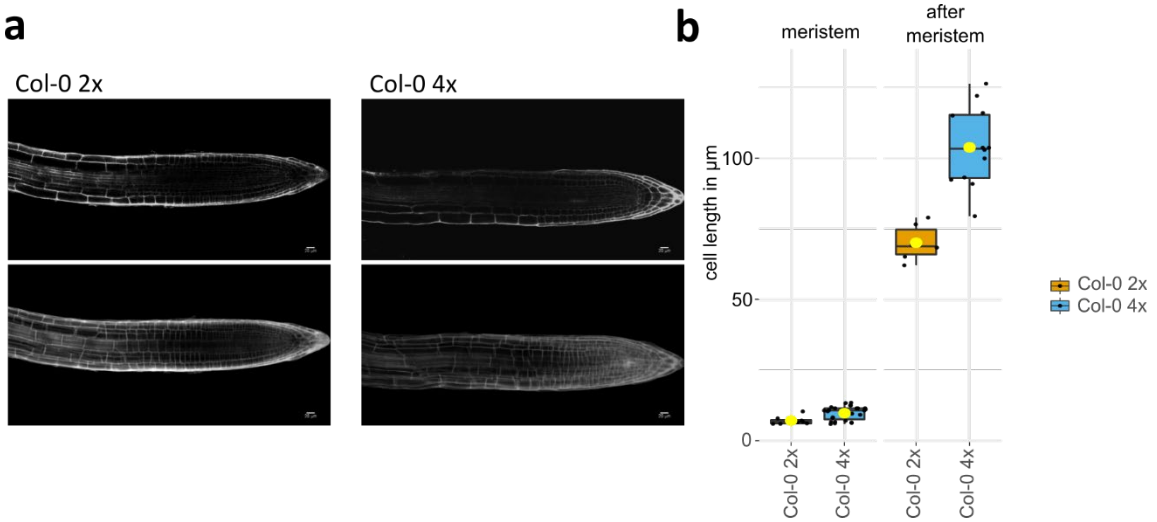

Supplemental Figure S7: Cell size of neo-tetraploids. a) Confocal image of propidium iodide-stained roots of diploid and neo-tetraploid plants grown on ½ MS, agar solidified, media. The size of meristematic and fully expanded cells were measured. Scale bar: 20µm **b)** General cell size difference in wild type Col-0 neo-tetraploids. Cells of different types are larger. The box plot shows the quantification of microscope images. The center line shows the median, box limits represent the first and third quartiles (the 25th and 75th percentiles), upper and lower whiskers extend to larges or smallest value no further than 1.5 inter-quartile range and dots beyond whiskers show outliers. Individual values are plotted as dots. n=6-26, Two way ANOVA shows significant differences for cell type ($p = 1.71E-13$) and genotype ($p = 0.000237$) and no interaction between the two ($p = 0.800364$, Sup Table 4).

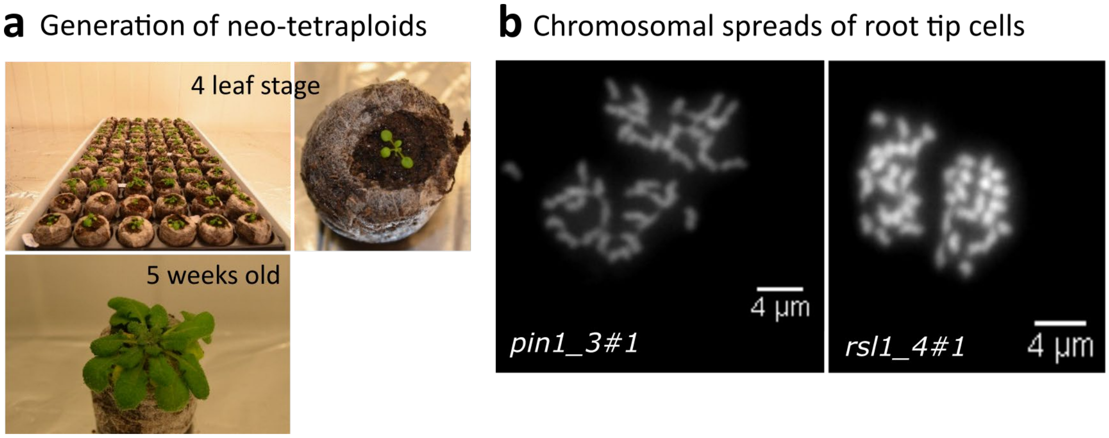

Supplemental Figure S8: Generation of new tetraploid lines. To generate new 4x lines plants of 4-leaf-stage were treated with colchicine and seeds collected of surviving plants. Among the progeny those with 4-branched trichomes were selected, grown for 5 weeks at which point a leaf was harvested for nuclei isolation. **a)** Pictures show Jiffi® growing plants in the 4-leaf stage just after colchicine treatment and a full setup of Jiffies® containing a set of 8 lines each growing on 13 Jiffies® in the WGD pipeline. Finally, a fully grown 5 week old plant in the 1st generation after WGD used to harvest nuclei before re-potting for seed generation. Nuclei, stained with PI were analyzed via flow cytometry. **b)** Flower buds were harvested and microscopic images of chromosomal spreads (Higgins et al., 2014) were taken. They show the 20 chromosomes of neo-tetraploid lines. This technique was used to verify the more high-throughput FACS analysis. Scale bar: 4µm

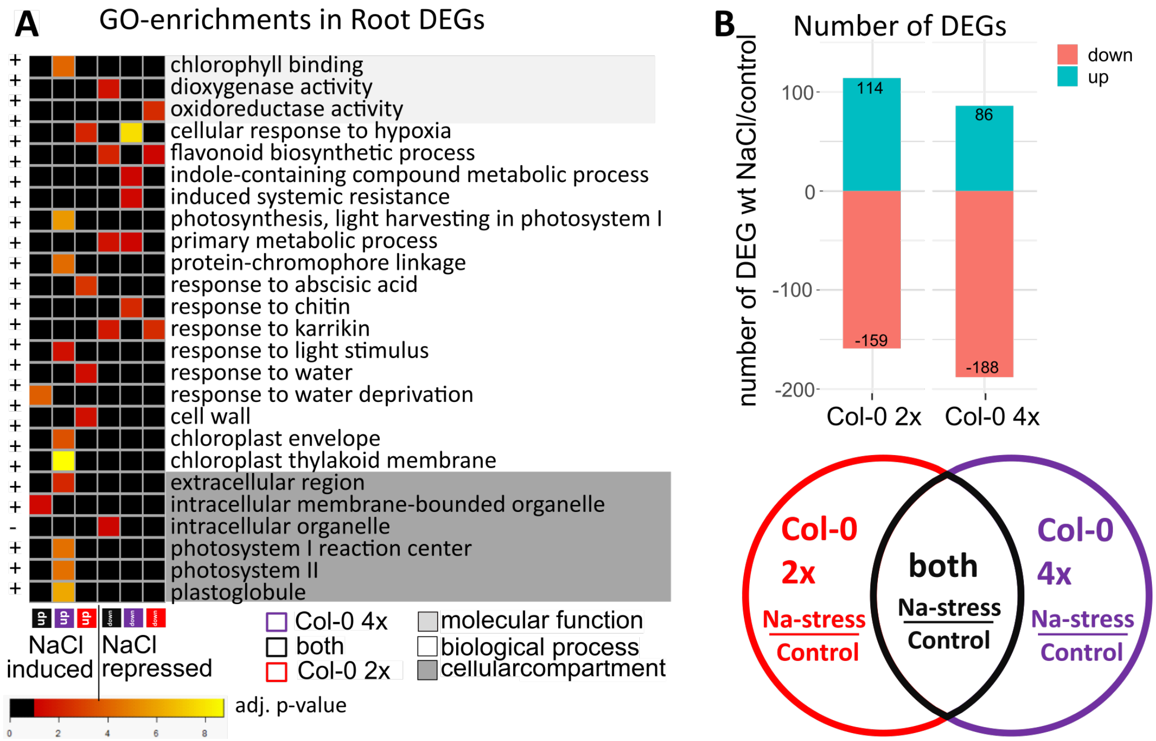

Supplemental Figure S9: PANTHER GO-enrichment analysis of the effect of Na on the root transcriptome. a) The heatmap shows p-values of 0.05 or lower, with yellow representing more significant -log (10) adjusted p-values for GO groups enriched in at least one of the 6 groups of genes. Analysis was done using PANTHER. Displayed are only the most defined terms or child terms. A full list can be found in Supplemental Table S5. Test Type: Fisher's Exact, Bonferroni correction for multiple testing, GO database release 2019-12-09. The groups of genes are annotated with different colours underneath the heatmap and the gene selection process is shown in **b)** Number of DEGs between control or Na stressed plants (either for diploids (2x) or for neo-tetraploids (4x)) are shown for root expressed genes. A Venn diagram shows the comparison between DEGs which was used for the GO analysis shown in Supplemental Figure S13 A. Genes were defined as being DEGs after Na stress in either diploids (red) or neo-tetraploids (purple) or both (black).

Abbreviations: *=oxidoreductase activity, acting on paired donors, with incorporation or reduction of molecular oxygen, **=chloroplast thylakoid membrane protein complex, transc.=transcription, resp.=response, membr.=membrane. Diploid (2x), neo-tetraploid (4x), GO= Gene Ontology, DEG= Differentially Expressed Genes

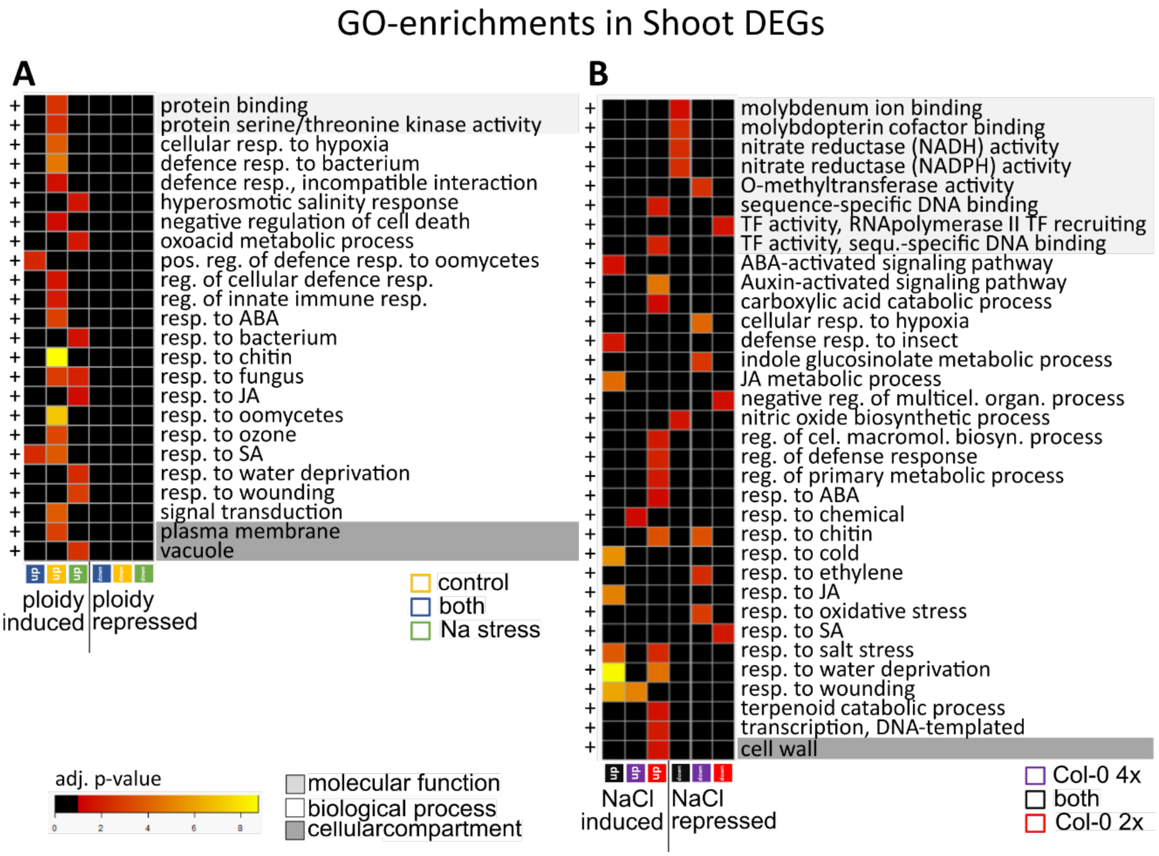

Supplemental Figure S10: PANTHER GO-enrichment analysis of shoot transcriptome. a-b) The heatmaps show p-values of 0.05 or lower, with yellow representing more significant -log (10) adjusted p-values for GO groups enriched in at least one of the 6 groups of genes. Analysis was done using PANTHER. Displayed are only the most defined terms or child terms. A full list can be found in Supplemental Table S5. Test Type: Fisher's Exact, Bonferroni correction for multiple testing, GO database release 2019-12-09. The groups of genes are annotated with different colors underneath the heatmap and the gene selection process is shown in Supplemental Figure S9.

Abbreviations: transc.=transcription, resp.=response, membr.=membrane. Diploid (2x), neo-tetraploid (4x), GO= Gene Ontology, DEG= Differentially Expressed Genes

Supplemental Table S1. ANOVA (Analysis of Variance) for Fig.s 1,3 and 4 as well as S3,4,7, and 10. The table shows the ANOVA results with the Degrees of freedom (DF) shows the numbers of each group, the Sum-of-squares (Sum Sq) quantifies how much variation is due to the fact that the difference between one factor are not the same for the other factor. The Mean squares (Mean Sq) are calculated by dividing the Sum Sq by the corresponding DF. The F value or F statistic is 0 if the H0 is true and measures the variation in data distribution of 2 groups by comparing Mean Sq. The Pr (>F) values shows the p-value for the F statistics. If the p-value is significant a code is added to each factor. The Details table shows the predictors, their estimates and Confidence Intervals (CI) and p-values (p), which are in bold when significant.

Supplemental Table S2. Raw ionomic data in ppm or µg/gDW (dry weight). For each sample, tissue and ploidy is given for each sample along with the element analyzed and plant weight where possible. Additionally samples are annotated by which Gene is affected by knockouts or tissue specific expression and Genotype which additionally differentiates between different alleles or independent ploidy events. The full list of genotypes and full explanation of the nomenclature can be found in Supplemental Table S6.

Supplemental Table S3. List of all differentially expressed genes. For expression analysis of plants grown on ¼ Hoagland medium in the first RNA-Seq experiment, DEGs were filtered for a fold change ≥2 and a diverge probability ≥0.8. For the second RNA-Seq experiment with plants grown on soil or on ½ MS medium, DEGs were filtered for a log2 fold change (L2 FC) of ≥ 0.5 and adjusted p-value < 0.05.

Supplemental Table S4. List of primers used in this study.

Supplemental Table S5: Full results of GO enrichment analysis of a subset of genes DE between wild type diploid and neo-tetraploids in roots either under control conditions, or under Na stress or both. See Supplemental Figure S1 to see a visual representation of the gene selections. The most detailed group (child term) is displayed graphically in a heatmap Fig. 5 and Supplemental Figure S9-10 as is highlighted here in the column "most detailed term". Each group of GO groups hierarchically connected to each other is marked in alternating yellow/grey and white background. GO number GSE180004 and GSE180818.

Supplemental Table S6. List of Arabidopsis lines used and generated for this study. Whenever possible several alleles were used for each gene of interest. If not possible it was attempted to generate 2 or more independent neo-tetraploid lines. Nomenclature was such: New lines were given a number/letter code which can be found in the "Comment" column for the original T-DNA line. For example, 6 for N653619 or *sur1-8*. Each plant which survived colchicine treatment was given incremental number such as 6-1, 6-8 being the 1st and the 8th plant to survive colchicine. Two seeds from the survivors were germinated and given a #1 or #2 addition making it 6-1#1 and 6-1#2 and 6-8#1 and 6-8#2. These lines were assessed for ploidy and neo-tetraploid lines kept for experimentation.

SI References

Afgan E, Baker D, van den Beek M, Blankenberg D, Bouvier D, Čech M, Chilton J, Clements D, Coraor N, Eberhard C, et al (2016) The Galaxy platform for accessible, reproducible and collaborative biomedical analyses: 2016 update. Nucleic Acids Res **44**: W3–W10

Benjamini Y, Yekutieli D (2001) The Control of the False Discovery Rate in Multiple Testing under Dependency. The Annals of Statistics **29**: 1165–1188

Campos ACAL, Dijk WFA van, Ramakrishna P, Giles T, Korte P, Douglas A, Smith P, Salt DE (2021) 1,135 ionomes reveal the global pattern of leaf and seed mineral nutrient and trace element diversity in Arabidopsis thaliana. The Plant Journal **106**: 536–554

Chao D-Y, Dilkes B, Luo H, Douglas A, Yakubova E, Lahner B, Salt DE (2013) Polyploids Exhibit Higher Potassium Uptake and Salinity Tolerance in Arabidopsis. Science **341**: 658–659

Danku JMC, Lahner B, Yakubova E, Salt DE (2013) Large-Scale Plant Ionomics. *In* FJM Maathuis, ed, Plant Mineral Nutrients: Methods and Protocols. Humana Press, Totowa, NJ, pp 255–276

Dietrich D, Pang L, Kobayashi A, Fozard JA, Boudolf V, Bhosale R, Antoni R, Nguyen T, Hiratsuka S, Fujii N, et al (2017) Root hydrotropism is controlled via a cortex-specific growth mechanism. Nature Plants **3**: 17057

Foreman J, Demidchik V, Bothwell JHF, Mylona P, Miedema H, Torres MA, Linstead P, Costa S, Brownlee C, Jones JDG, et al (2003) Reactive oxygen species produced by NADPH oxidase regulate plant cell growth. Nature **422**: 442–446

Forieri I, Sticht C, Reichelt M, Gretz N, Hawkesford MJ, Malagoli M, Wirtz M, Hell R (2016) Systems analysis of metabolism and the transcriptome in Arabidopsis thaliana roots reveals differential co-regulation upon iron, sulfur and potassium deficiency. Plant, Cell & Environment n/a-n/a

Gierth M, Mäser P, Schroeder JI (2005) The Potassium Transporter AtHAK5 Functions in K+ Deprivation-Induced High-Affinity K+ Uptake and AKT1 K+ Channel Contribution to K+ Uptake Kinetics in Arabidopsis Roots. Plant Physiol **137**: 1105–1114

Guo W, Tzioutziou NA, Stephen G, Milne I, Calixto CP, Waugh R, Brown JWS, Zhang R (2020) 3D RNA-seq: a powerful and flexible tool for rapid and accurate differential expression and alternative splicing analysis of RNA-seq data for biologists. RNA Biology 1–14

Hampton CR, Bowen HC, Broadley MR, Hammond JP, Mead A, Payne KA, Pritchard J, White PJ (2004) Cesium Toxicity in Arabidopsis. Plant Physiology **136**: 3824–3837

Henry IM, Dilkes BP, Young K, Watson B, Wu H, Comai L (2005) Aneuploidy and Genetic Variation in the Arabidopsis thaliana Triploid Response. Genetics **170**: 1979–1988

Higgins JD, Wright KM, Bomblies K, Franklin C (2014) Cytological techniques to analyze meiosis in Arabidopsis arenosa for investigating adaptation to polyploidy. Front Plant Sci. doi: 10.3389/fpls.2013.00546

Hong J-P, Takeshi Y, Kondou Y, Schachtman DP, Matsui M, Shin R (2013) Identification and Characterization of Transcription Factors Regulating Arabidopsis HAK5. Plant Cell Physiol **54**: 1478–1490

Lamesch P, Berardini TZ, Li D, Swarbreck D, Wilks C, Sasidharan R, Muller R, Dreher K, Alexander DL, Garcia-Hernandez M, et al (2011) The Arabidopsis Information Resource (TAIR): improved gene annotation and new tools. Nucl Acids Res gkr1090

Lê S, Josse J, Husson F (2008) **FactoMineR** : An *R* Package for Multivariate Analysis. J Stat Soft. doi: 10.18637/jss.v025.i01

Menand B, Yi K, Jouannic S, Hoffmann L, Ryan E, Linstead P, Schaefer DG, Dolan L (2007) An Ancient Mechanism Controls the Development of Cells with a Rooting Function in Land Plants. Science **316**: 1477–1480

Nieves-Cordones M, Alemán F, Martínez V, Rubio F (2014) K+ uptake in plant roots. The systems involved, their regulation and parallels in other organisms. Journal of Plant Physiology **171**: 688–695

Paredes SH, Gao T, Law TF, Finkel OM, Mucyn T, Teixeira PJPL, González IS, Feltcher ME, Powers MJ, Shank EA, et al (2018) Design of synthetic bacterial communities for predictable plant phenotypes. PLOS Biology **16**: e2003962

Patro R, Duggal G, Love MI, Irizarry RA, Kingsford C (2017) Salmon provides fast and bias-aware quantification of transcript expression. Nat Methods **14**: 417–419

Ragel P, Ródenas R, García-Martín E, Andrés Z, Villalta I, Nieves-Cordones M, Rivero RM, Martínez V, Pardo JM, Quintero FJ, et al (2015) The CBL-Interacting Protein Kinase CIPK23 regulates HAK5-Mediated High-Affinity K+ uptake in Arabidopsis roots. Plant Physiol 2863–2873

Ramakrishna P, Duarte PR, Rance GA, Schubert M, Vordermaier V, Vu LD, Murphy E, Barro AV, Swarup K, Moirangthem K, et al (2019) EXPANSIN A1-mediated radial swelling of pericycle cells positions anticlinal cell divisions during lateral root initiation. PNAS **116**: 8597–8602

Roppolo D, De Rybel B, Tendon VD, Pfister A, Alassimone J, Vermeer JEM, Yamazaki M, Stierhof Y-D, Beeckman T, Geldner N (2011) A novel protein family mediates Casparian strip formation in the endodermis. Nature **473**: 380–383

Schindelin J, Arganda-Carreras I, Frise E, Kaynig V, Longair M, Pietzsch T, Preibisch S, Rueden C, Saalfeld S, Schmid B, et al (2012) Fiji: an open-source platform for biological-image analysis. Nat Methods **9**: 676–682

Seifert GJ, Barber C, Wells B, Dolan L, Roberts K (2002) Galactose Biosynthesis in Arabidopsis. Current Biology **12**: 1840–1845

Sigg CD, Buhmann JM (2008) Expectation-maximization for sparse and non-negative PCA. Proceedings of the 25th international conference on Machine learning - ICML '08. ACM Press, Helsinki, Finland, pp 960–967

Soneson C, Love MI, Robinson MD (2016) Differential analyses for RNA-seq: transcript-level estimates improve gene-level inferences. F1000Res. doi: 10.12688/f1000research.7563.2

Wu F-H, Shen S-C, Lee L-Y, Lee S-H, Chan M-T, Lin C-S (2009) Tape-Arabidopsis Sandwich - a simpler Arabidopsis protoplast isolation method. Plant Methods **5**: 16

Yi K, Menand B, Bell E, Dolan L (2010) A basic helix-loop-helix transcription factor controls cell growth and size in root hairs. Nat Genet **42**: 264–267

Young L-S, Harrison BR, U.M. NM, Moffatt BA, Gilroy S, Masson PH (2006) Adenosine Kinase Modulates Root Gravitropism and Cap Morphogenesis in Arabidopsis. Plant Physiology **142**: 564–573

Yu Z, Haage K, Streit VE, Gierl A, Ruiz RAT (2009) A large number of tetraploid Arabidopsis thaliana lines, generated by a rapid strategy, reveal high stability of neo-tetraploids during consecutive generations. Theor Appl Genet **118**: 1107–1119
